# The Lipid Paradox as a Metabolic Checkpoint and Its Therapeutic Significance in Ameliorating the Associated Cardiovascular Risks in Rheumatoid Arthritis Patients

**DOI:** 10.3390/ijms21249505

**Published:** 2020-12-14

**Authors:** Tapan Behl, Ishnoor Kaur, Aayush Sehgal, Gokhan Zengin, Ciprian Brisc, Mihaela Cristina Brisc, Mihai Alexandru Munteanu, Delia Carmen Nistor-Cseppento, Simona Bungau

**Affiliations:** 1 Chitkara College of Pharmacy, Chitkara University, Punjab 140401, India; ishnoorkaur7@gmail.com (I.K.); aayushsehgal00@gmail.com (A.S.); 2 Department of Biology, Faculty of Science, Selcuk University Campus, 42130 Konya, Turkey; biyologzengin@gmail.com; 3 Department of Medical Disciplines, Faculty of Medicine and Pharmacy, University of Oradea, 410073 Oradea, Romania; brisciprian@gmail.com (C.B.); briscristina@yahoo.com (M.C.B.); mihaimunteanual@yahoo.com (M.A.M.); 4 Department of Psycho-Neuroscience and Recovery, Faculty of Medicine and Pharmacy, University of Oradea, 410073 Oradea, Romania; delia_cseppento@yahoo.com; 5 Department of Pharmacy, Faculty of Medicine and Pharmacy, University of Oradea, 410028 Oradea, Romania

**Keywords:** rheumatoid arthritis, cardiovascular disorders, lipid paradox, inflammatory burden, LDL-C, HDL-C, lipoproteins, atherosclerosis, DMARDs

## Abstract

While the most common manifestations associated with rheumatoid arthritis (RA) are synovial damage and inflammation, the systemic effects of this autoimmune disorder are life-threatening, and are prevalent in 0.5–1% of the population, mainly associated with cardiovascular disorders (CVDs). Such effects have been instigated by an altered lipid profile in RA patients, which has been reported to correlate with CV risks. Altered lipid paradox is related to inflammatory burden in RA patients. The review highlights general lipid pathways (exogenous and endogenous), along with the changes in different forms of lipids and lipoproteins in RA conditions, which further contribute to elevated risks of CVDs like ischemic heart disease, atherosclerosis, myocardial infarction etc. The authors provide a deep insight on altered levels of low-density lipoprotein cholesterol (LDL-C), high-density lipoprotein cholesterol (HDL-C) and triglycerides (TGs) in RA patients and their consequence on the cardiovascular health of the patient. This is followed by a detailed description of the impact of anti-rheumatoid therapy on the lipid profile in RA patients, comprising DMARDs, corticosteroids, anti-TNF agents, anti-IL-6 agents, JAK inhibitors and statins. Furthermore, this review elaborates on the prospects to be considered to optimize future investigation on management of RA and treatment therapies targeting altered lipid paradigms in patients.

## 1. Introduction

Rheumatoid arthritis (RA) is considered to be an autoimmune disorder which is prevalent in about 0.5–1% of the general population [1,2], with significant risks of comorbidities, disabilities and fatigue [3], along with cardiovascular disorders (CVDs), and long-term impact on socioeconomic and personal paradigms [4]. Even though no exact cause is known, the disease is considered to occur as a result of a combination of epigenetic, genetic and environmental factors, and the progression of the disorder is considered to be initiated years before appearance of clinical signs and symptoms [5]. Therefore, several studies have established the importance of early diagnosis to provide treatment at early stages, which has proved to be beneficial, along with prognostic markers for remission [6,7].

A bioclinical and chemical aspect, comprising anti-citrullinated peptide antibodies (ACPA), has been incorporated by the 2010 American College of Rheumatology/European League of Rheumatism criteria for RA [8]. The identification of ACPA and rheumatoid factor (RF) autoantibodies, of different isotypes, in the circulation, are considered to be significant markers in early diagnosis of RA, prior to the clinical manifestation of the disorder [9]. RA patients exhibit metabolic alterations, which may elevate morbidity and mortality risks in the patients [10]. Such metabolic alterations are identified by evaluation of the basal metabolic rate (BMR) of RA patients, which is reported to elevate by 8%, unlike in the healthy individual [11].

Furthermore, significant alterations in the blood lipid are also reported in RA patients [12], which may depict increased cardiovascular (CV) risks in such patients. Furthermore, RA elevates the risk of CVDs by 50% (approximately) as compared to the general population [13,14], and CVDs are the leading cause of death in patients with RA [13,15,16,17,18,19,20]. The risk for myocardial infarction (MI) has been reported to be enhanced by 2-fold, compared to control groups, as observed in large retrospective RA investigations [15,21]. RA patients are more susceptible to ischemic heart disease, heart failure and CV mortalities, and also the pattern of CVDs in patients with RA is revealed to be different from that of general population [20].

Type 2 diabetes, hypertension and smoking are considered to be traditional risk factors of CVDs [22,23], which play a significant role in elevating the mortality rate in RA patients [24,25]. The elevation in CV risks is primarily driven by inflammatory responses related to RA [13,26]. Thus, enhanced inflammatory processes in RA patients are associated with atherosclerotic events, along with systemic inflammatory responses, which are responsible for adverse alterations in CV risk factors [26,27,28,29,30].

The RA-associated lipid paradox is related to an excessive inflammatory burden in RA patients, in which an inverse association is observed between cholesterol (a significant CV risk factor in general population) and CV risks in RA patients (untreated) [31,32]. On the other hand, inhibition of inflammatory events related to RA coincides with certain elevations in lipid concentration, along with amelioration in CV events [33]. The significance of minimizing CV risks in RA is considered to be fundamental as per the recommendations laid by the European League against Rheumatism (EULAR) for coronary heart diseases (CHDs) management associated with RA [33]. Evaluation of CV risks in RA patients is recommended on an annual basis [13].

Based on the metabolic alterations observed in RA patients and the role of these changes in inducing CV, the aim of this review is to provide a detailed overview of the lipid paradox, along with its role in developing CV risks in such subjects; we also focused on the impact of anti-rheumatoid therapies in the lipid scenario associated with RA. The authors highlight the relationship between elevated risks of CV, the inflammatory burden and altered lipid profiles in patients with RA. Significant information is also provided on the importance of lipid alterations associated with RA in serving as appropriate therapeutic targets. Over 350 references were searched and 221 of them were cited as supporting claims of the current study.

## 2. Metabolic Frontiers in Rheumatoid Arthritis and Their Therapeutic Significance

RA is an inflammatory disorder of the immune system, characterized by the production of self-antibodies such as ACPA, RF and anti-carbamylated protein antibodies (anti-CarP). [34]. This is accompanied by chronic inflammation of the synovial tissue and hyperplasia, damage to the bone and cartilage as well as systemic complexities, significantly related to the lungs, brain or CV system, which pose a fundamental threat to the socioeconomic balance and unmet needs [34,35]. RA is associated with progressive therapeutic advancement with conventional treatment therapies and disease modifying anti-rheumatic drugs (DMARDS); however, these agents have been able to provide optimum response in only 60% of the RA patients [36].

Presently, the predictive biomarkers evaluating the prognostic approach, treatment efficacy and resistance to therapy, consisting of RF, C-reactive protein (CRP), ACPA and erythrocyte sedimentation rate (ESR), remain insufficient from a clinical perspective [5,37]. The immune system intolerance is marked as a primary event in RA pathogenesis, which is followed by inflammation of the joint [5,38], which is most likely to take place at the extracellular site [39]. The events (such as infiltration of leukocytes, production of new vasculature and elevated expression levels of chemokines and adhesion molecules) result in enhanced migration of leukocytes to the site of inflammation [34]. Furthermore, improper formation of lymphatic vessels restricts cell retreat, along with activation of fibroblasts, resulting in inflammation of the synovial tissue [40]. The joint resident and immune system cells compete for nutrients due to limited nutrient availability, at a rate exceeding that of their formation, thus elevating the metabolic requirement [41,42,43,44,45]. All these events significantly induce changes in the immune responses, resulting in immune intolerance, leading to inflammation and autoimmunity [34].

**Figure 1:**
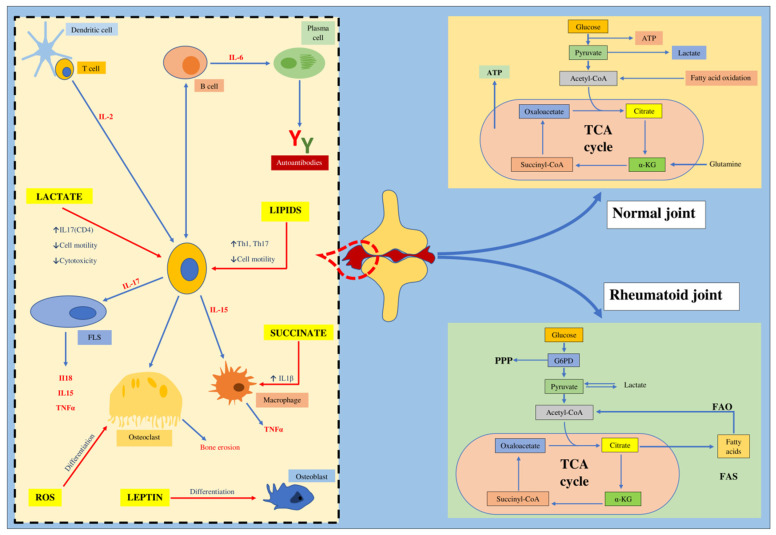
The inflammatory portfolio in RA (rheumatoid arthritis) synovium and impaired metabolic processes. Legend: IL-2,6,17,15, Interleukin-2,6,17,15; CD4, cluster of differentiation 4; Th1,7, T-helper cells; FLS, fibroblast-like synoviocytes; TNF-α, tumor necrosis factor alpha; ROS, reactive oxygen species; IL-1ß, interleukin-1 beta; ATP, adenosine triphosphate; TCA, tricarboxylic acid cycle; α-KG, alpha-ketoglutaric acid; G6PD, glucose-6 phosphate dehydrogenase; PPP, pentose phosphate pathway; FAS, fatty acid synthase; CoA, coenzyme A.

The investigation of metabolic intermediates and end products, relative to the functions of the immune cells, is a progressing area of research currently, which has been referred to as immunometabolism [46]. Certain molecules like acetyl-CoA, succinate, fumarate and lactate, function as signaling molecules, establishing significant associations between metabolic processes and inflammatory and immune responses (Figure 1) [34].

Urine and serum sample metabolomics, based upon nuclear magnetic resonance (NMR) spectroscopy, has identified greater levels of lactate and 3-hydroxybutyrate among the metabolites in a group of RA patients, unlike the control group, where the serum metabolic profile was evaluated using 1-dimensional (1) H-NMR spectroscopy [47]. Furthermore, mitochondrial DNA (mtDNA) mutations and production of reactive oxygen species (ROS) were reported to be present in greater amounts in patients with RA, as compared to osteoarthritis fibroblast-like synoviocytes (FLS), when 50 subjects with inflammatory arthritis went through arthroscopy and synovial tissue biopsies, where their synovial fluid was clinically evaluated [48]. Random mutation capture assay (RMCA) and specific cell fluorescent probes were employed for quantification of ROS, mitochondrial membrane potential (MMP), mass and mutagenesis. The elevated mitochondrial mutations are related to the inflammation of the synovial membrane, depicting a direct association between mutations and prime proinflammatory pathways [48]. Unlike T cells, RA FLS depict elevation in glycolytic metabolism under conditions of metabolic stress [49]. Similarly, lipid metabolism is also found to play an important role in regulation of the functions of the immune cells, according to the recent studies [50], which has brought the lipid mediators into light, as significant therapeutic targets in various allergic and autoimmune disorders [51]. Table 1 enlists various metabolic targets associated with RA.

Certain specific transcription factors function as metabolic sensors and regulate numerous anabolic and catabolic pathways, like 5′ AMP-activated protein kinase (5′ AMPK), i.e., a redox sensor [52], which regulates various metabolic activities, such as mitochondrial biogenesis, glucose uptake, cellular functions and lipid metabolism [34].

The experimental arthritis was found to be suppressed as a result of therapeutic activation of AMPK, like methotrexate-mediated AMPK-dependent pathway stimulation, which is depicted to exert protective effects against inflammation [53,54]. The activation of AMPK is dependent upon myristoylation, and the RA T cells exhibit a flaw in the function of N-myristoyl transferase (NMT), which disables the AMPK activation event and enables the activation of mammalian target of rapamycin1 (mTOR1) signaling, promoting differentiation of pro-inflammatory Th1 and Th17 [34]. The investigations regarding loss of function of NMT1 were found to induce in vivo and in vitro inflammatory responses, whereas, on the contrary, excessive expression levels of NMT1 were found to restore the activation of AMPK and inhibit inflammation of the synovial tissue [55].

Furthermore, an indirect activator of AMPK, metformin (anti-diabetic drug), has been reported to curb the disease progression in mouse arthritic models [56] by inhibiting the mTOR pathway, elevating autophagic flux and suppressing nuclear factor-kappa B (NF-Κb)- induced production of inflammatory cytokines [53]. The environmental signals, cellular functions and nutrient availability is regulated by both mTOR and AMPK [57,58]. However, activation of mTOR is marked with aging of the cells (senescence), therefore, rapamycin (inhibitor of mTOR complex 1) has been recognized as an agent in treating autoimmune, degenerative and hyperproliferative disorders [59]. The potential of mTOR to synergize bioenergetics, nutrient supply and functions of T cells makes it a reliable therapeutic target in the suppression of abnormal differentiation of T cells during initial RA phases [59].

Similarly, several drugs affecting the metabolic signaling pathways are used to treat RA patients, such as glucocorticoids, which block the fructose 2, 6-biphosphate (glycolytic enzyme) in tymocytes in rats and modulate the rate of respiration in the peripheral blood mononuclear cells of rheumatic patients [60]. Regulation of purine and pyrimidine nucleotide metabolism is the prime mechanism of anti-inflammatory actions rendered by methotrexate [61].

Treatment with anti-tumor necrosis factor-α (anti-TNF-α) agents and janus kinase (JAK) inhibitors, like tofacitinib, mitigate glycolysis in the synovium of RA patients [62]. Tocilizumab (anti-IL-6 blocker) curbs the oxidative stress (OS) conditions in leukocytes of RA patients [63]. Therefore, numerous drugs have been identified in RA treatment by affecting the metabolic signaling pathways to mitigate inflammatory responses in both in vitro and in vivo models of RA [42,53,64,65]. Similarly, evaluating the role of lipid metabolism in RA can prove to be effective for the development of suitable therapeutic possibilities and associate lipid metabolism abnormalities to RA [34]. Figure 1 depicts the inflammatory processes in synovial tissue of RA patients, along with multiple metabolic alterations.

## 3. Cardiovascular Risk and Inflammatory Burden in RA

One of the major CV risk factors is inflammation, which is evident by data revealing lower CV risks in RA as a result of mitigated inflammatory responses [66,67,68,69,70]. Employment of traditional equations to assess CV risk factors, such as systemic coronary risk evaluation (SCORE) models and Framingham, are considered to underestimate this risk in RA patients, as they are not able to evaluate the role of systemic inflammation and its effect on lipid profiles in patients [24,71,72,73]. The occurrence and pathogenesis of CVDs and atherosclerosis in the general population are significantly affected by inflammation, according to evidence-based data [74,75,76].

Numerous pro-inflammatory molecular entities, like fibrinogen, CRP and cytokines, aid in the regulation of this process, as per the data obtained from epidemiological studies [77,78,79]. RA patients are marked with elevated levels of inflammatory molecules and cytokines which promote dysfunction of endothelial cells and structural vessel deformities, alongside induction of other CV risk factors, like insulin resistance, alterations in lipid levels and oxidative stress [80,81,82]. Furthermore, many investigational studies have established an important link between CVD risk and inflammatory processes in RA [32,83,84,85,86,87,88,89,90]. Inflammation plays a significant role in all the stages of atherosclerosis [16,82,91]. RA and atherosclerosis are associated with common inflammatory processes and the events resulting in the inflammation of synovial tissue are similar to those in the case of unstable atherosclerosis [78,82,91].

Inflammation is related to an inverse association between CV risks and lipid pattern in RA [32,66,92]. This kind of link has also been reported in the post-surgical time span, where an inverse relationship has been noted between cholesterol levels and IL-6 enhancement [93]. Numerous investigational studies have revealed elevation in the level of lipids, with a significant amelioration in RA progression after therapy with anti-inflammatory agents [94]. These outcomes reveal that the traditional elucidation of the lipid portfolio to carry out prediction of general CV risks may be expressed by prevalence of disease in RA patients [32,66].

The mechanisms concerned with the effect of inflammatory responses on lipid alterations are yet to be fully understood but might account for reticuloendothelial system (RES) suppression and abbreviated formation of low-density lipoprotein (LDL) [66]. There is a possibility of impairment of cholesterol trafficking in the liver due to overproduction of acute phase reactants (APR) under an elevated inflammatory burden [33]. Moreover, LDL and oxidized LDL (oxLDL) uptake is promoted by C-reactive protein (CRP), followed by LDL deposition and elevation in its uptake by liver cells [95,96]. Both quantitative and qualitative alterations in lipoproteins account for the inflammatory burden in RA [97]. High density lipoprotein (HDL) exhibits athero-protective and anti-inflammatory functions, facilitating reverse cholesterol transport (RChT) from the blood circulation to the liver and hampering oxidation of LDL [98].

CV risk-propagating pathological events might impair such protective actions [99,100,101,102,103,104]. Certain changes were found to exist in the composition of HDL, which was isolated from the patients with RA, according to the proteomic studies, along with the loss of reverse cholesterol transport and anti-inflammatory action [97,105,106]. Some studies depict anti-inflammatory HDL to be a more sensitive marker for CVDs, as compared to absolute HDL [33]. For instance, studies related to torcetrapib and dalcetrapib (cholesterol ester transfer protein inhibitors) report 30% to 70% elevation in the levels of circulating HDL, yet no cardioprotective action was revealed [107,108]. Dalcetrapib (600 mg/day) or placebo were administered to 15,871 patients with acute coronary syndrome, resulting in 4–11% elevation in HDL-C levels in the placebo group, as well as a 31–40% rise in the dalcetrapib group. There was a negligible effect observed on LDL-C levels by dalcetrapib administration. Unlike the placebo, there was no change in the risk of primary end point or total mortality. Moreover, the mean systolic blood pressure (BP) was reported to be 0.6 mm Hg greater and the level of median C-reactive protein was found to be 0.2 mg/L higher in the case of dalcetrapib administration, unlike the placebo [107]. Therefore, such studies depict the significance of both quantitative and qualitative alterations in the assessment of lipid profiles in RA patients [109,110,111].

## 4. An Overview of Lipids and Lipoproteins

Dyslipidemia refers to alterations in plasma lipid levels. Atherogenicity occurs as a result of elevated levels of cholesterol and triglycerides (TGs) in plasma. The elevated expression of lipids is significantly associated with the enhanced production of lipids and alleviated removal or absorption. On the other hand, abbreviated expression of lipids may occur as a result of reduced production of lipids and/or enhanced clearance [112]. The lipids, primarily TGs and cholesterol, are water insoluble forms, which are transported by blood, and depending upon their association with proteins are known as lipoproteins, which are complex entities comprised of cholesterol ester and TGs containing a central core [113]. These particles are surrounded by a shell comprised of phospholipids, apolipoproteins and free cholesterol, facilitating the functions and formation of lipids [112].

On the basis of composition of lipids, size and apolipoproteins, the lipoproteins are divided into the following categories: chylomicrons and chylomicron remnants. The very low-density lipoprotein (VLDL), high density lipoprotein (HDL), intermediate density lipoprotein (IDL), lipoprotein-a (Lp-a) and low-density lipoprotein (LDL) are all considered as chylomicron remnants by the authors; however, this is a basic biochemical misconception because of the different and type-specific apolipoproteins characterizing the different groups of lipoproteins, respectively ApoB-48 vs. ApoA-I and ApoA-II vs. ApoB-100. The VLDL, LDL, IDL and Lp-a are considered to be pro-atherogenic, whereas HDL is considered to be anti-atherogenic [112].

**Figure 2:**
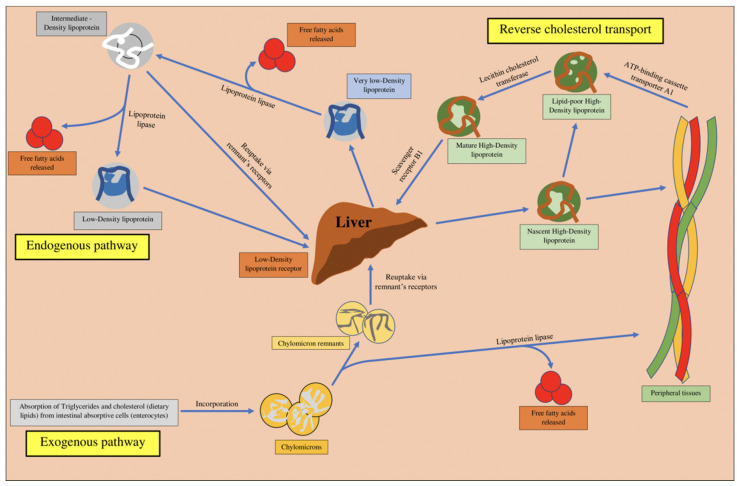
Schematic illustration of exogenous and endogenous pathways of cholesterol.

Two types of pathway, mainly the exogenous and endogenous pathway, act independently and promote the transportation of dietary lipids in the blood, promoting hepatic and peripheral movement of lipids from the small intestine (Figure 2).

The triglycerides and cholesterol esters in diet are emulsified by bile acids secreted by the liver, for hydrolysis by lipases in the intestine, followed by re-esterification of these fats to triglycerides and cholesterol esters, which are then packed into chylomicrons. Chylomicrons are large lipoproteins with density < water, which enter the blood and are rapidly cleared by lipoprotein lipase. This enzyme hydrolyzes triglycerides to free fatty acids which are utilized for production of energy, while the excess is stored as triglycerides in the adipose tissue. The remaining “chylomicron remnant” undergoes hepatic clearance. This part of metabolism of lipoproteins is named as the exogenous pathway [114].

The endogenous pathway comprises synthesis and secretion of very low-density lipoproteins (VLDL), which are degraded by lipoprotein lipase, resulting in production of intermediate density protein (IDL), followed by formation of low-density lipoprotein (LDL). In the presence of the LDL receptor, LDL gets stored in the liver and peripheral tissues, otherwise it undergoes oxidation and gets attached to the scavenger receptor on the macrophages [114]. LDL comprises the maximum amount of cholesterol that is in circulation. Furthermore, the high-density lipoproteins (HDL) play a significant role in reverse cholesterol transport from the peripheral tissues to the liver. HDL possesses anti-atherogenic, anti-thrombotic, antioxidant, anti-apoptotic and anti-inflammatory properties, and is abundant in cholesterol and phospholipids [115].

The excess of cholesterol is removed from the peripheral tissues to the liver by a reverse transport mechanism, referred to as reverse cholesterol transport (RChT) [112]. The exogenous lipoprotein pathway is initiated by administration of dietary lipids into intestinal chylomicron, which undergo further metabolism in the muscles and adipose tissue with the help of lipoprotein lipase enzyme, resulting in production of free fatty acids (FFSs) and chylomicron remnants, which then exhibit hepatic uptake. The endogenous pathway of lipoprotein is initiated in the liver, with the formation of VLDL, followed by metabolism of TGs (contained in VLDL) in the muscles and adipose tissue, with the help of lipoprotein lipase enzyme, resulting in the production of FFAs and IDL [112,116,117]. The IDL formed is transformed into LDL, which is taken up by the LDL receptor, mainly contained primarily in liver. The RChT is initiated by the formation of nascent HDL by the intestinal and hepatic tissue and ATP-binding cassette transporter A1 (ABCA1) facilitates the transportation of cholesterol and phospholipids in the cells from the peripheral tissue to nascent HDL, resulting in the production of mature HDL (by lecithin cholesterol acyltransferase, LCAT), which can further acquire more cellular cholesterol with the help of ABCG1 and class-B-scavenger receptor B1 (SR-B1). Cholesterol is transported to the liver, which is enabled by interaction between HDL and hepatic SR-B1, or by cholesterol transportation to LDL, with the help of cholesterol ester transfer protein (CETP) [115,118,119,120]. It can only exit the body by biliary excretion, once it enters the liver.

Depending upon the size, LDL can be grouped as large LDL, which is named pattern A, while small LDL is named as pattern B. The latter are related to CVDs, due to easy penetration ability of small particles into the target cell endothelium. Oxidized LDL (oxLDL) is a term used for LDL particles comprising oxidative modified structural components. Therefore, as a result of the attack by the free radicals, both protein components of LDL and lipids can undergo oxidation in the vascular wall [112]. The oxLDL particles are not recognized by the LDL receptor, which hinders the normal metabolism of LDL particles, resulting in atherosclerosis, which explains the atherogenicity of oxLDL [115,118,119,120].

Normally, HDL plays a significant role in oxLDL inhibition and efflux of cholesterol from the foam cells in the vessel wall [110]. There is no valid clarification of anti-inflammatory and atherogenic actions of HDL, but it has been reported that the functions exhibited by HDL are dependent upon its protein composition. Some amount of LDL is oxidized, in the case of elevated formation or reduced clearance of lipids, resulting in the formation of oxLDL, which is phagocytosed by macrophages, resulting in the formation of foam cells, followed by their deposition on the walls of the artery, facilitating atherosclerotic plaque formation. This establishes the significance of cholesterol efflux via RChT pathway, in order to maintain cholesterol homeostasis in the cells and promote prevention of atherosclerosis and reduction of toxic cholesterol expression in each cell [112].

Apolipoproteins are produced in the intestine and liver and contribute to metabolism of lipids, by functioning as lipoprotein receptor ligands and co-factors for lipid metabolism-associated enzymes. One of the primary components of the structure of HDL is apolipoprotein A-1 (Apo A-1), which is synthesized in the liver and accounts for 70% of the HDL structure, whereas, on the contrary, apolipoprotein A-2 (Apo A-2) accounts for 20% of the HDL structure. Other apolipoproteins, produced in the intestine, are referred to as apolipoprotein B-48 (Apo B-48), which is a significant structural component of chylomicrons and chylomicron remnants, and apolipoprotein B-100 (Apo B-100) which is primarily synthesized in the liver and forms an important structural component of VLDL, LDL and IDL [115,118,119,120]. Figure 2 illustrates endogenous and exogenous lipoprotein pathways, along with reverse cholesterol transport.

## 5. PUFAS and Phospholipids in RA Patients

The prime six types of lipids, as per the Lipid Maps, have been assessed in the plasma samples of healthy subjects where >500 species of lipids were recognized and samples were collected from 100 healthy subjects, representing common ethnicities in the USA, and stored post overnight fasting. Sterols like cholesterol was found to be present in heavy amounts in the samples, while prenols and diacylglycerols presented in limited amounts. The free fatty acids, triglycerides, sphingolipids and glycerophospholipids, were found in intermediate quantities in the sample. Polyunsaturated fatty acids (PUFAS) were also identified in the sample, with arachidonic acid and linoleic acid in abundance, along with anti-inflammatory fish oil derivatives, primarily, docosahexaenoic acid (DHA) and eicosapentaenoic acid (EPA) [121,122]. The lipoxygenase (LOX) metabolites, like 5-HETE, and cyclooxygenase (COX) metabolites, like 15-deoxy-prostaglandin D2 (PGD2), were also detected, along with lipid mediator oxylipins [123]. PUFAS are dietary fatty acids, where n-3 PUFA EPA and DHA are anti-inflammatory while n-6 PUFA AA is considered to proinflammatory. The phospholipids comprise long chain fatty acids, like AA, EPA and DHA, which constitute the cell membrane [121]. Various investigations have been performed using n-3 supplements or fish oil derivatives, such as one where the authors observed significant variations in orally administered DHA and EPA (fish oil supplement) doses, after evaluating 23 studies. The time period of the investigations varied between 1–13 months and oils such as olive, paraffin and corn were used as placebo controls. The average sample size was found to be 20–30 patients per group. Various studies had methodological defects and no meta-analysis was carried out. The n-3 PUFAS administration was associated with swelling in joints, pain and morning stiffness, however, the overall effect was prudent [124]. Another study reported similar levels of free fatty acids in RA patients and healthy individuals [125], depicting no significant quantitative variation in the level of free fatty acids in the diseased state. In one study, the serum samples of RA patients were found to exhibit lower ratios of phosphatidylcholine (PC)/lysophosphatidylcholine (LPC), unlike in healthy subjects [126], and portrayed greater activity of poly-lactic acid in patients with RA, which could enhance the level of free fatty acids that can further be metabolized into bioactive lipids. However, not only fatty acid precursors but also COX-generated lipid mediators (PGD2 and PGE2) were found to be present in serum samples of RA patients in significant amounts. [121,127]. The most abundant phospholipid found in the synovial fluid of RA patients was PC, followed by sphingomyelins and LPC, which were found in greater concentration in RA patients as compared to controls [128]. The ratio of PC/LPC was greater in the synovial fluid of RA patients, unlike controls, which is the opposite to what was found in the serum [128]. The synovial fluid of RA patients was investigated for eicosanoids, where PGE2, PLA2 and COX were found to be greater in the synovium of RA patients [129,130]. Furthermore, more anti-inflammatory prostaglandins, like PGD2, and its metabolite 15-deoxy-PGJ2, along with leukotrienes, were also reported to be present in the RA patients [129]. Moreover, anti-inflammatory LOX products were also found to be present in the synovial fluid of RA patients, comprising anti-inflammatory and pro-resolving mediators lipoxin A4 (LXA4) as well as m-3 PUFA DHA derivatives, such as resolving D5 and maresin 1 [131]. This investigation was carried out on only five subjects and the role of pro-resolving lipids in RA was not investigated, however, they were recognized as suitable therapeutic agents for chronic inflammatory disorders due to their potential immune modulatory functions [132].

## 6. Lipid Metabolism in RA

RA patients show curbed LDL-C, HDL-C and TC levels, which are enhanced by therapies targeting inflammatory processes associated with RA [32]. A U-shaped association is proposed between CV risk and lipid pattern, in a so-called “RA lipid paradox”, where the patients with reduced LDL-C levels have greater risk of developing CVDs compared to those with moderate levels of LDL-C [92]. The abbreviated levels of HDL-C in patients with RA facilitates enhanced atherogenic index of TC/HDL-C ratio [92,133]. The early stages of RA are associated with an atherogenic lipid profile [133,134].

The concentration of lipids is inversely related to the inflammatory markers in RA patients [135]. Even though the data available regarding the influence of treatment on HDL-C are inconsistent, the levels of HDL-C are considered to be consistent relative to inflammatory alterations [106,136,137]. Furthermore, alteration in the level of lipids is more closely related to CRP changes than those of disease activity score 28 (DAS28) for RA, comprising clinical and laboratory data for evaluation of disease activity [138]. The definite cause for changes in the RA-associated lipid profile is yet to be fully understood, however, studies show that such a lipid paradox is due to inflammatory processes and elevated cholesterol catabolism [111,112]. The expression of LDL and SR-B1 receptors is enhanced by proinflammatory cytokines such as IL-6 and TNF-α, which lead to elevated liver uptake of LDL and biliary secretion of cholesterol [139,140], resulting in reduced levels of circulating LDL. This process was depicted by studies investigating metabolism of cholesterol by exhibiting lipid labeling with stable isotopes [141]. The fractional catabolic rate (FCR) was employed in two investigations to evaluate the catabolic clearance, where the first one reported greater levels of cholesterol ester FCR in patients with RA, as compared to those belonging to the control group, which demonstrated greater cholesterol ester catabolism, resulting in alleviated cholesterol levels in patients [112]. The FCR for cholesterol ester was abbreviated and the level of cholesterol was enhanced following treatment with tofacitinib [142]. Further, in one study the FCR of LDL was found to be in the hyper-catabolic range compared to the general population, which was decreased to the level similar to that of the general population after treatment with tocilizumab [143]. Moreover, oxidation is another mechanism which results in reduced levels of circulating LDL, where studies show that patients with RA exhibit a greater number of autoantibodies against mildly oxidized LDL, elaborating the alleviated action of lipoprotein-associated phospholipase A2 [144,145].

The extent of inflammation is related to the effect of LDL on CVD risk when the erythrocyte sedimentation rate exceeds 30 mm/h [89]. Greater inflammation in RA is represented by high CRP, which is related to elevated CV risk [133,146]. Furthermore, investigations have depicted that inflammatory markers, like CRP and ESR, are related to the intima-media thickness [147,148]. In one study, the effects of canakinumab (IL-1 monoclonal antibody) were investigated and a 15% reduction in CV events was reported as a result of reduced inflammation [149]. Moreover, the antioxidant capacity of HDL is reported to be affected by inflammatory responses. The anti-inflammatory ability of HDL is disturbed in animals [103,104] and humans [111] as their ability to facilitate cholesterol clearance from atherosclerotic plaques is lost and it becomes pro-atherogenic [12,150]. The damaged pro-inflammatory HDL is marked by reduced antioxidant factors [101], along with elevation of pro-inflammatory proteins [104]. In addition, it comprises enhanced lipid hyperoxide levels [111], resulting in ameliorated cholesterol efflux [151] and reduced oxLDL preventive ability [152]. The levels of HDL-associated antioxidant enzyme, paraoxonase (PON), are abbreviated in RA patients as compared to controls [99], whereas an investigation revealed that alterations in the antioxidant function of HDL were observed, expressed by elevated PON, following therapy with TNF-α inhibitor [153]. Additionally, Watanabe et al. revealed the presence of an altered proteome in pro-inflammatory HDL in RA patients, comprising elevated levels of acute-phase proteins, like serum α amyloid, fibrinogen and haptoglobin, as well as proteins of the complement system [97]. The levels of secretory phospholipase A2 were found to be reduced along with serum α amyloid (SAA) during tocilizumab therapy with modifications of the composition of lipoproteins [154]. Therefore, all these investigations and events support the lipid paradox in RA, along with great C risks in patients with RA, mostly associated with lipid qualitative aspects (primarily the HDL) which become pro-atherogenic after losing the anti-atherogenic action. The inflammatory processes are reduced by therapeutic treatment of RA patients; however, the levels of LDL-C, HDL-C and TC are elevated, which is not related to increased CV events [92].

Short chain fatty acids (SCFAs) have been reported to carry out various functions of CD4^+^ cells by regulating the actions of histone deacetylases (HDAC) [155] and peroxisome proliferator-activated receptor (PPAR) signaling pathway [156]. Lipid metabolism is also crucial for T cell activation and proliferation, and elevation of sterol regulatory element binding protein (SREBP) levels [34]. The genetic inactivation promotes SREBP loss, which is harmful for the T cells, which exhibit post-activation clonal expansion [157]. Elevated fatty acid synthesis (FAS) has been revealed from T cells isolated from RA patients, resulting in enhanced tissue invasiveness [34]. Furthermore, 6-phosphofructo-2-kinase/fructose-2,6-biphosphatase 3 enzyme (PFKFB3) deficiency-mediated glycolytic flux propagates a shunt towards anabolic use of glucose (elevated PPP and FAS) and enhanced levels of podosome scaffold adapter protein TKS5 (SH3PXD2A), which contributes to the formation of protrusions in the cell membrane [64,158]. The cytoplasmic lipid droplets are accumulated as a result of increased FAS, which is fundamental for the functions of T cells, like growth and proliferation of cells, as well as transformation of naïve to memory T cell [34]. The locomotion of T cells can be regained as well as inflammation and tissue invasiveness can be minimized in diabetic severe combined immunodeficiency (SCID) mice, without obesity imbedded with synovial tissue of humans, by restoring pyruvate levels [34]. Additionally, tissue inflammation was curbed and the number of infiltrating T cells, receptor activation of nuclear factor kappa-B ligand (RANKL^+^) and interferon-gamma^+^ (INF-γ^+^) T cells was reduced [64]. The differentiation of Th17 cells was regulated by de novo production of fatty acids [159]. Sorafen A showed that in vitro inhibition of acetyl-CoA carboxylase (ACC) results in disrupted Th17 differentiation, which promotes Foxp3^+^ Treg cell differentiation instead of T-helper 17 (Th17) cells [159]. It has been also revealed the fact that when lactate is present in amounts as compared to those analyzed in the synovial tissue, the CD4^+^ T cells are found to elevate the de novo production of fatty acids, resulting in enhanced levels of IL-17 and curbed cell motility [160]. However, all such events were restored after treatment with FAS inhibitors which alleviated the NADPH levels induced by lactate [160]. Cholesterol metabolism regulates the CD4^+^ T cell-regulated anti-inflammatory response in humans, whereas de novo production of fatty acids plays a fundamental role in functions of effector CD4^+^ T cells [161]. A specific hindrance in the immune system resolution and a remarkable reduction in the levels of c-Maf/IL-10 has been shown by 25-hydroxycholesterol and atorvastatin-mediated inhibition of cholesterol biosynthesis during INF-γ^+^ to IL-10^+^ switching [161].

Lipid metabolism in RA and osteoarthritis FLS has been altered as per the metabolomics data. The synovial tissues in RA FLS were found to be greatly expressed with choline and choline-like transporter, CTL1 and CTL2, where the former exhibits high affinity and the latter exhibits low affinity [162,163]. Inhibition of their functions lead to the death of FLS cells [164]. These outcomes were aided by the results of positron emission tomography (PET) scanning with ^11^C-choline, depicting enhanced uptake in affected joints [164].

## 7. Effect of Anti-Rheumatic Therapies on Lipid Profile in RA

The administration of biological agents facilitates a treat-to-target approach contributing to greater understanding of CVD-associated risks in RA. Investigations provide a suitable amount of evidence related to the role of csDMARDs and bDMARDs, however, more clinical research is required in this regard [112]. Chen et al. depicted potential effects of biological therapy on insulin resistance and lipid profiles in RA patients, where they demonstrated an inverse relationship between LDL-C and disease progression, as well as a positive association between insulin resistance and DAS28 [165]. Similarly, insulin resistance exhibits a positive relationship with IL-6 and TNF levels [166].

The patients receiving biological therapeutic treatment were considered to exhibit lower insulin resistance as compared to those who were not [166]. The DMARDs and other biological therapies aid in the significant improvement of lipid profiles, along with curbed CV risk factors [67,167,168,169]; four out of ten RA patients fail to attain the desired targets for lipids, hypertension and diabetes diagnoses [170,171].

### 7.1. DMARDs and Corticosteroids

Corticosteroids provide symptomatic relief from pain in RA and also aid in amelioration of inflammatory events. However, they are associated with certain adverse effects, mainly elevation in CV risk factors such as hypertension and carotid plaque formation [172,173]. The risk of heart problems is twice more in the case of administration of high dose steroids, as compared to the cases without steroid administration, whereas the low dose, short-term corticosteroids alter the plasma lipid levels primarily by enhancing the levels of HDL-C [174].

Traditional DMARDs (like methotrexate, hydroxychloroquine etc.) exhibit protective actions against CV risks in RA, out of which methotrexate is considered to be the most significant drug [33]. Furthermore, csDMARDS have been considered to affect the lipid profile according to various studies conducted [136,150,168,175,176,177,178]. A drug used for malaria, hydroxychloroquine (HCQ), can be employed for the treatment of mild RA and has been considered to enhance the levels of HDL, either by ameliorating activity of the disease or by directly influencing the metabolism of lipids [168]. Methotrexate is presently employed as a first line drug in RA treatment and has been considered to reduce CV events by 21%, as per a meta-analysis report [175]. Additionally, methotrexate exhibits an athero-protective role by facilitating RChT and minimizing the formation of foam cells in THP-1 macrophages [176]. However, no alterations were reported in the lipid profile in some clinical studies after methotrexate administration, when the drug was administered alone or combined with other bDMARDs [177,178,179,180].

Certain studies report a significant decline in the levels of LDL-C, TC and HDL-C as well as the capacity of cholesterol efflux [136,181,182]. Georgiadis et al. depicted enhanced TC and HDL-C expression, along with a reduced TC/HDL-C ratio, after a year-long treatment of RA patients with a methotrexate–prednisolone combination [133,134]. A strong inverse association between HDL-C and CRP levels was reported, without any alteration in serum LDL-C levels [112].

### 7.2. Anti TNF-α Agents

TNF is an important cytokine in chronic inflammation, which influences lipid metabolism, the function of the endothelial cells and insulin resistance [183,184]. Anti-TNF therapy has been reported to reduce inflammation and expression of levels of ESR and CRP [185,186]. Moreover, in combination with methotrexate or DMARDs, it also regulates the lipoprotein spectrum, and has been considered to ameliorate the CV risks in RA patients [68,69,70]. Certain studies have found that anti-TNF therapy has been associated with 54% reduction in CV risks [187]. This therapeutic approach has been found to regulate factors related to atherosclerotic CV risks in RA patients, such as mitigation of endothelial dysfunction [188,189,190,191], improved insulin sensitivity [184] and enhanced HDL anti-oxidative capacity [153]. Numerous investigations depict a significant elevation in the serum apoB and LDL-C levels following treatment with anti-TNF-αagents [185,192]. On the other hand, various other studies reveal a neutral impact of infliximab drug on lipid pattern, due to no alterations in LDL-C, TC/HDL-C or TGs/HDL-C levels during treatment [193,194,195]. TNF-α inhibitors are considered to affect the levels of TC and HDL-C, without exerting any effect on the atherogenic index in RA patients [94,196,197]. Furthermore, no significant relationship is reported between combined therapy of anti-TNF-α agents, steroids and csDMARDs with lipid profiles of RA patients [198]. Therefore, the resultant efficacy of infliximab on management of CV diseases may be associated with other factors, like improvement in insulin resistance and arterial stiffness; however, further studies are essential to support this hypothesis [199,200].

Published data have depicted elevated TG levels and an alleviated apolipoprotein B/A ratio as a result of long-term treatment with TNF inhibitors [94]. Moreover, the risk of acute coronary syndrome was lowered in RA patients receiving TNF inhibitors, unlike those who were biologically naïve, according to a national Swedish cohort study, which elaborated upon the future benefits of inhibiting this cytokine [201]. Mostly, older RA patients are associated with changes in lipid profiles and elevated CV risks, however, juvenile patients with idiopathic arthritic problems reported improvement in lipid profiles after treatment with etanercept (a TNF blocker) [202].

### 7.3. Anti-IL-6 Agents

An anti-IL-6 monoclonal antibody, tocilizumab, hinders the signaling process of IL-6 and shows potential therapeutic significance in RA. IL-6 is considered to influence metabolism of lipids by promoting uptake of lipids by VLDLR induction and elevating hepatic and adipose tissue lipolysis as well as abbreviating lipid production in the liver [203]. The serum TG, TC and HDL-C levels are reported to be enhanced by anti-IL-6 agents, as per numerous study outcomes [204,205]. It is noteworthy that the effect on atherogenic index is inconsistent, however, as various investigations demonstrate 15–20% elevation in LDL-C levels [205]. A MEASURE study (a randomized, parallel group, open-label, multicenter investigation to assess tocilizumab effects on vaccination in RA patients administered with methotrexate) also demonstrated the elevated LDL-C levels as a result of tocilizumab treatment, which also modified the HDL particles to anti-inflammatory composition [154]. Anti-atherogenic small and medium particles were reported to be enhanced with administration of tocilizumab. Moreover, the investigation also showed fundamental alterations in HDL-associated serum amyloid A (SAA) levels, paraoxonase 1 and secreted group 2A phospholipase A_2_ with tocilizumab treatment. The mono therapeutic response of tocilizumab and adalimumab (anti-TNF) was comparatively evaluated in methotrexate-intolerant RA patients in a double-blind adalimumab actemra (ADACTA) study (phase 4), where the results depicted elevated LDL, CRP, DAS28 (28-joint DAS) and ESR in 6 months in greater number of patients in the tocilizumab treatment group, as compared to the adalimumab treatment group [206]. Tocilizumab also exhibited greater abbreviation in Clinical Disease Activity Index (CDAI), which does not constitute an APR component [206]. Tocilizumab was also found to improve the insulin resistance in RA patients in a TOWARD (tocilizumab in combination with traditional DMARD therapy) meta-analysis investigation [207,208].

Moreover, the rates of myocardial infarction were numerically decreased with administration of tocilizumab as compared to the controls, in a double-blind phase of five core phase 3 tocilizumab trials, whereas evaluation of long-term safety of tocilizumab depicted a stable prevalence of CV events over time with tocilizumab treatment [209,210].

### 7.4. JAK Inhibitors

These agents hinder the JAK–STAT signaling pathway, resulting in reduced immune response and RA remission. A dual JAK1-JAK3 inhibitor, tofacitinib, upregulated the levels of HDL-C and LDL-C to about 14% and 21% within a year of treatment, in a phase 3, double-blind, placebo controlled, parallel group study of 6 months conducted on 611 subjects, who were assigned randomly in a ratio 4:4:1:1 to 5 mg of the drug two times a day, 10 mg drug two times a day, placebo for 3 months, followed by 10 mg of drug two times a day [211]. This elevation, in deadlocked comparison between JAK inhibitors and adalimumab, was greater than that observed post treatment with anti-TNF-α agents [165,212]. Ameliorated cholesterol water FCR might facilitate elevated cholesterol levels during JAK inhibitor therapy in patients with RA [142]. The US FDA has approved tofacitinib (JAK inhibitor) as a RA medication [112]. The levels of LDL and HDL were found to be significantly elevated with tofacitinib administration in a phase 3 study, as compared to adalimumab at 3 months [213]. The LDL and TCh levels were reported to be reduced to baseline levels by administration of combination of tofacitinib and atorvastatin in a phase 2 study [214].

### 7.5. Other Agents

A chimeral monoclonal antibody, rituximab, has been employed in RA treatment, where it has been considered to improve atherogenic index and lipid profile, as per certain studies [215,216]. Rituximab was administered to 55 women with RA and no CVDs, and the following parameters were assessed before and after 6 months of therapy: HDL-C, LDL-C, plasma total cholesterol (TC), serum C-reactive protein, RF IgM, triglycerides, AS (by digital volume pulse contour analysis), DAS 28-ESR and common cIMT (by high-resolution B-mode carotid ultrasound [215]. The patients were grouped under two categories based upon whether the results were good following 6 months of rituximab therapy or whether no response was observed. TC was elevated by 9%, HDL-C by 23%, AI was decreased by 14%, along with SI and RI by 57% and 24%, as a result of effective rituximab therapy [215]. In another study, intravenous administration of two infusions of 1000 mg rituximab was carried out in five women with RA, and branchial FMD and ccIMT was evaluated using high-resolution B-mode ultrasound, along with determination of HDL-C, TC and LDL-C levels. The results depicted elevated FMD, reduced TC (by 3–11%) and increased HDL-C levels (by 14–35%). Potential effects were exerted on endothelial dysfunction as well as plasma TC and HDL-C levels by two infusions of rituximab [216]. However, on the other hand, Mathieu et al. depicted no improvement in arterial stiffness, atherogenicity index or LDL-C in a study conducted on 33 non-responding RA patients to anti-TNF treatment therapies [217]. Therefore, further investigations are necessary to assess the definite effects of rituximab on CV risks in RA patients. Furthermore, the lipid profile can also be improved by statins, along with prevention of CV risks in general and RA patients [218,219,220]. These agents promoted small relative and absolute reduction in LDL-C levels in RA patients, as compared to those without RA [166]. However, these agents are not used much in clinical practice [221].

## 8. Future Directions and Conclusions

This review emphasizes the significance of the lipid paradox in RA and details the requirement for future research to deeply understand the lipid portfolio to facilitate a “treat-to-target” approach to reduce RA-associated CV risks. The RA metabolomics studies employ NMR and MS to extricate RA from other inflammatory conditions and controls [166]. Future prospects would more likely facilitate collaboration between metabolomic data of RA and human metabolic networks, like Recon 2 [166]. The metabolic pathways, enzymes, transcription factors and metabolites, which are altered in patients with RA, are identified as significant therapeutic targets in RA management, as per the immune-metabolic studies. Many currently used drugs target the metabolic pathways in RA. However, there is a need to develop specific therapeutic approaches targeting RA-associated metabolic pathways. For instance, inflammatory responses have been found to be ameliorated in both in vitro and in vivo models of arthritis, as a result of specifically targeting metabolic processes in RA [64,65]. Furthermore, metabolic intermediates like succinate and lactate are also becoming a potential possibility [34]. Animal models have proved to be quite effective in therapeutic screening during preclinical investigations; however, certain treatment therapies, which have exhibited safe and effective results in preclinical assessment, have failed to depict optimum results in clinical investigations in humans. Therefore, greater understanding of human immunology and identification of animal models similar to clinical models is required. Gender has been also found to exert a significant effect on RA immunometabolism, as RA prevalence is greater in women as compared to men [34]. This can be somewhat explained by the impact of sex hormones on regulation of the immune system, and their relationship with genetic and environmental aspects [34], but this still needs further investigation. Mass spectroscopy and NMR are considered to be significant tools for predicting the altered pathogenic pathways in RA [34].

In the future, the results might prove to be effective in identifying the risk of developing atherosclerosis in RA patients. Moreover, future studies should strive to differentiate RA conditions on the basis of the stage of the disorder, outcome and therapeutic response, by using specific metabolic signatures. Single cell RNA-seq and advanced RNS-seq techniques can be used as promising tools in cellular profiling in the future [34]. In addition, new biomarkers can be identified and novel therapeutic approaches, targeting impaired metabolic signaling pathways, can be developed, without hindering immune system homeostasis, with the help of single cell metabolomic analysis.

This review details the impact of an impaired lipid portfolio in RA and its relationship with occurrence of CV risks in patients. Numerous forms of metabolic checkpoints are highlighted in the text, out of which the review has emphasized the RA-associated lipid paradox. This is followed by an overview of CV risk and inflammatory burden associated with the lipid profile in RA patients. The authors provide a detailed overview of the pathways and processes comprising lipids and lipoproteins, along with the role of lipid metabolism in RA, which paves a way for understanding the impact of anti-rheumatoid therapeutic approaches on the lipid profile of RA patients.

Therefore, in the current review, the authors aim to provide a significant opportunity to the researchers to correlate RA-associated lipid profile with elevated CV risks, and to facilitate recognition of the impaired lipid paradox in RA as an appropriate therapeutic possibility, to reduce the RA-associated events along with related CVDs, thereby proposing an optimistic approach in the management of RA.

## Figures and Tables

**Table 1:** Metabolic targets in treatment of RA.

Cell Associated with RA	Abnormal Metabolic Process		Effective Therapeutic Targets
	Increased	Decreased	
Fibroblasts	Glycolysis; Lipid	-	HK2; GLUT1; PFKFB3; Choline
T cell	Lipid; PPP	Glycolysis	FASN; PFKFB3; AMPK/mTOR; G6PD; Lactate
Dendritic cells	Glycolysis	-	iNOS; HK2; mTOR
Macrophages/monocytes	TCA; Glycolysis	(AMPK)	HIF; Lactate; PKM2; Succinate

**Legend**: (TCA, tricarboxylic acid cycle; AMPK—5′ AMP-activated protein kinase; HK2, hexokinase-2; GLUT1, glucose transporter 1; PFKFB3, 6-phospho-fructo-2-kinase/fructose-2,6-biphosphatase 3 enzyme; FASN, fatty acid synthase; mTOR, mammalian target of rapamycin; G6PD, glucose-6-phosphate dehydrogenase; iNOS, inducible nitric oxide synthase; HIF, hypoxia inducible factors; PKM2, pyruvate kinase M2.

## References

[1] Humphreys J.H., Verstappen S.M., Hyrich K.L., Chipping J.R., Marshall T., Symmons D.P. (2013). The incidence of rheumatoid arthritis in the UK: Comparisons using the 2010 ACR/EULAR classification criteria and the 1987 ACR classification criteria. Results from the Norfolk Arthritis Register. Ann. Rheum. Dis..

[2] Symmons D., Turner G., Webb R., Asten P., Barrett E., Lunt M., Scott D., Silman A. (2002). The prevalence of rheumatoid arthritis in the United Kingdom: New estimates for a new century. Rheumatology.

[3] Dougados M., Soubrier M., Antunez A., Balint P., Balsa A., Buch M.H., Casado G., Detert J., El-Zorkany B., Emery P. (2014). Prevalence of comorbidities in rheumatoid arthritis and evaluation of their monitoring: Results of an international, cross-sectional study (COMORA). Ann. Rheum. Dis..

[4] Grøn K.L., Ornbjerg L.M., Hetland M.L., Aslam F., Khan N.A., Jacobs J.W., Henrohn D., Rasker J.J., Kauppi M.J., Lang H.C. (2014). The association of fatigue, comorbidity burden, disease activity, disability and gross domestic product in patients with rheumatoid arthritis. Results from 34 countries participating in the Quest-RA program. Clin. Exp. Rheumatol..

[5] McInnes I.B., Schett G. (2011). The pathogenesis of rheumatoid arthritis. N. Engl. J. Med..

[6] Demoruelle M.K., Deane K.D. (2012). Treatment strategies in early rheumatoid arthritis and prevention of rheumatoid arthritis. Curr. Rheumatol. Rep..

[7] Van der Linden M.P., le Cessie S., Raza K., van der Woude D., Knevel R., Huizinga T.W., van der Helm-van Mil A.H. (2010). Long-term impact of delay in assessment of patients with early arthritis. Arthritis Rheum..

[8] Aletaha D., Neogi T., Silman A.J., Funovits J., Felson D.T., Bingham C.O., Birnbaum N.S., Burmester G.R., Bykerk V.P., Cohen M.D. (2010). 2010 Rheumatoid arthritis classification criteria: An American College of Rheumatology/European League Against Rheumatism collaborative initiative. Arthritis Rheum..

[9] Rantapää-Dahlqvist S., de Jong B.A., Berglin E., Hallmans G., Wadell G., Stenlund H., Sundin U., van Venrooij W.J. (2003). Antibodies against cyclic citrullinated peptide and IgA rheumatoid factor predict the development of rheumatoid arthritis. Arthritis Rheum..

[10] Toms T.E., Symmons D.P., Kitas G.D. (2010). Dyslipidaemia in rheumatoid arthritis: The role of inflammation, drugs, lifestyle and genetic factors. Curr. Vasc. Pharmacol..

[11] Metsios G.S., Stavropoulos-Kalinoglou A., Nevill A.M., Douglas K.M., Koutedakis Y., Kitas G.D. (2008). Cigarette smoking significantly increases basal metabolic rate in patients with rheumatoid arthritis. Ann. Rheum. Dis..

[12] Myasoedova E., Crowson C.S., Kremers H.M., Fitz-Gibbon P.D., Therneau T.M., Gabriel S.E. (2010). Total cholesterol and LDL levels decrease before rheumatoid arthritis. Ann. Rheum. Dis..

[13] Peters M.J., Symmons D.P., McCarey D., Dijkmans B.A., Nicola P., Kvien T.K., McInnes I.B., Haentzschel H., Gonzalez-Gay M.A., Provan S. (2010). EULAR evidence-based recommendations for cardiovascular risk management in patients with rheumatoid arthritis and other forms of inflammatory arthritis. Ann. Rheum. Dis..

[14] Aviña-Zubieta J.A., Choi H.K., Sadatsafavi M., Etminan M., Esdaile J.M., Lacaille D. (2008). Risk of cardiovascular mortality in patients with rheumatoid arthritis: A meta-analysis of observational studies. Arthritis Rheum..

[15] Solomon D.H., Karlson E.W., Rimm E.B., Cannuscio C.C., Mandl L.A., Manson J.E., Stampfer M.J., Curhan G.C. (2003). Cardiovascular morbidity and mortality in women diagnosed with rheumatoid arthritis. Circulation.

[16] De Groot L., Posthumus M.D., Kallenberg C.G., Bijl M. (2010). Risk factors and early detection of atherosclerosis in rheumatoid arthritis. Eur. J. Clin. Investig..

[17] Gullick N.J., Scott D.L. (2011). Co-morbidities in established rheumatoid arthritis. Best Pract. Res. Clin. Rheumatol..

[18] Meune C., Touzé E., Trinquart L., Allanore Y. (2010). High risk of clinical cardiovascular events in rheumatoid arthritis: Levels of associations of myocardial infarction and stroke through a systematic review and meta-analysis. Arch. Cardiovasc. Dis..

[19] Gremese E., Ferraccioli G. (2011). The metabolic syndrome: The crossroads between rheumatoid arthritis and cardiovascular risk. Autoimmun. Rev..

[20] Gabriel S.E. (2008). Cardiovascular morbidity and mortality in rheumatoid arthritis. Am. J. Med..

[21] Semb A.G., Kvien T.K., Aastveit A.H., Jungner I., Pedersen T.R., Walldius G., Holme I. (2010). Lipids, myocardial infarction and ischaemic stroke in patients with rheumatoid arthritis in the Apolipoprotein-related Mortality RISk (AMORIS) Study. Ann. Rheum. Dis..

[22] Stoicescu M., Csepento C., Mutiu G., Bungau S. (2011). The role of increased plasmatic renin level in the pathogenesis of arterial hypertension in young adults. Rom. J. Morphol. Embryol..

[23] Gheorghe G., Toth P.P., Bungau S., Behl T., Ilie M., Stoian A.P., Bratu O.G., Bacalbasa N., Rus M., Diaconu C.C. (2020). Cardiovascular Risk and Statin Therapy Considerations in Women. Diagnostics.

[24] Dessein P.H., Joffe B.I., Veller M.G., Stevens B.A., Tobias M., Reddi K., Stanwix A.E. (2005). Traditional and nontraditional cardiovascular risk factors are associated with atherosclerosis in rheumatoid arthritis. J. Rheumatol..

[25] Boyer J.F., Gourraud P.A., Cantagrel A., Davignon J.L., Constantin A. (2011). Traditional cardiovascular risk factors in rheumatoid arthritis: A meta-analysis. Jt. Bone Spine.

[26] Ku I.A., Imboden J.B., Hsue P.Y., Ganz P. (2009). Rheumatoid arthritis: Model of systemic inflammation driving atherosclerosis. Circ. J..

[27] Weinblatt M.E., Kuritzky L. (2007). RAPID: Rheumatoid arthritis. J. Fam. Pract..

[28] Situnayake R.D., Kitas G. (1997). Dyslipidemia and rheumatoid arthritis. Ann. Rheum. Dis..

[29] Del Rincón I.D., Williams K., Stern M.P., Freeman G.L., Escalante A. (2001). High incidence of cardiovascular events in a rheumatoid arthritis cohort not explained by traditional cardiac risk factors. Arthritis Rheum..

[30] Sattar N., McInnes I.B. (2005). Vascular comorbidity in rheumatoid arthritis: Potential mechanisms and solutions. Curr. Opin. Rheumatol..

[31] National Cholesterol Education Program (NCEP) (2002). Expert Panel on Detection, Treatment of High Blood Cholesterol in Adults (Adult Treatment Panel III), Third Report of the National Cholesterol Education Program (NCEP) Expert Panel on Detection, Evaluation, and Treatment of High Blood Cholesterol in Adults (Adult Treatment Panel III) final report. Circulation.

[32] Myasoedova E., Crowson C.S., Kremers H.M., Roger V.L., Fitz-Gibbon P.D., Therneau T.M., Gabriel S.E. (2011). Lipid paradox in rheumatoid arthritis: The impact of serum lipid measures and systemic inflammation on the risk of cardiovascular disease. Ann. Rheum. Dis..

[33] Choy E., Ganeshalingam K., Semb A.G., Szekanecz Z., Nurmohamed M. (2014). Cardiovascular risk in rheumatoid arthritis: Recent advances in the understanding of the pivotal role of inflammation, risk predictors and the impact of treatment. Rheumatology.

[34] Pucino V., Certo M., Varricchi G., Marone G., Ursini F., Rossi F.W., De Paulis A., Mauro C., Raza K., Buckley C.D. (2020). Metabolic checkpoints in rheumatoid arthritis. Front. Physiol..

[35] Makkar R., Behl T., Bungau S., Kumar A., Arora S. (2020). Understanding the Role of Inflammasomes in Rheumatoid Arthritis. Inflammation.

[36] Humby F., Lewis M., Ramamoorthi N., Hackney J.A., Barnes M.R., Bombardieri M., Setiadi A.F., Kelly S., Bene F., DiCicco M. (2019). Synovial cellular and molecular signatures stratify clinical response to csDMARD therapy and predict radiographic progression in early rheumatoid arthritis patients. Ann. Rheum. Dis..

[37] Dennis G., Holweg C.T., Kummerfeld S.K., Choy D.F., Setiadi A.F., Hackney J.A., Haverty P.M., Gilbert H., Lin W.Y., Diehl L. (2014). Synovial phenotypes in rheumatoid arthritis correlate with response to biologic therapeutics. Arthritis Res. Ther..

[38] Smolen J.S., Aletaha D., McInnes I.B. (2016). Rheumatoid arthritis. Lancet.

[39] Tracy A., Buckley C.D., Raza K. (2017). Pre-symptomatic autoimmunity in rheumatoid arthritis: When does the disease start?. Semin. Immunopathol..

[40] Croft A.P., Campos J., Jansen K., Turner J.D., Marshall J., Attar M., Savary L., Wehmeyer C., Naylor A.J., Kemble S. (2019). Distinct fibroblast subsets drive inflammation and damage in arthritis. Nature.

[41] Patella F., Schug Z.T., Persi E., Neilson L.J., Erami Z., Avanzato D., Maione F., Hernandez-Fernaud J.R., Mackay G., Zheng L. (2015). Proteomics-based metabolic modeling reveals that fatty acid oxidation (FAO) controls endothelial cell (EC) permeability. Mol. Cell. Proteom..

[42] Biniecka M., Canavan M., McGarry T., Gao W., McCormick J., Cregan S., Gallagher L., Smith T., Phelan J.J., Ryan J. (2016). Dysregulated bioenergetics: A key regulator of joint inflammation. Ann. Rheum. Dis..

[43] Tsokos G.C. (2016). Metabolic control of arthritis: Switch pathways to treat. Sci. Transl. Med..

[44] Yang Z., Shen Y., Oishi H., Matteson E.L., Tian L., Goronzy J.J., Weyand C.M. (2016). Restoring oxidant signaling suppresses proarthritogenic T cell effector functions in rheumatoid arthritis. Sci. Transl. Med..

[45] Zhou J., Chen J., Hu C., Xie Z., Li H., Wei S., Wang D., Wen C., Xu G. (2016). Exploration of the serum metabolite signature in patients with rheumatoid arthritis using gas chromatography-mass spectrometry. J. Pharm. Biomed. Anal..

[46] Pearce E.L., Poffenberger M.C., Chang C.H., Jones R.G. (2013). Fueling immunity: Insights into metabolism and lymphocyte function. Science.

[47] Young S.P., Kapoor S.R., Viant M.R., Byrne J.J., Filer A., Buckley C.D., Kitas G.D., Raza K. (2013). The impact of inflammation on metabolomic profiles in patients with arthritis. Arthritis Rheum..

[48] Harty L.C., Biniecka M., O’Sullivan J., Fox E., Mulhall K., Veale D.J., Fearon U. (2012). Mitochondrial mutagenesis correlates with the local inflammatory environment in arthritis. Ann. Rheum. Dis..

[49] Falconer J., Murphy A.N., Young S.P., Clark A.R., Tiziani S., Guma M., Buckley C.D. (2018). Review: Synovial cell metabolism and chronic inflammation in rheumatoid arthritis. Arthritis Rheumatol..

[50] Cucchi D., Camacho-Muñoz D., Certo M., Pucino V., Nicolaou A., Mauro C. (2019). Fatty acids—From energy substrates to key regulators of cell survival, proliferation and effector function. Cell Stress.

[51] Marone G., Galdiero M.R., Pecoraro A., Pucino V., Criscuolo G., Triassi M., Varricchi G. (2019). Prostaglandin D(2) receptor antagonists in allergic disorders: Safety, efficacy, and future perspectives. Expert Opin. Investig. Drugs.

[52] Shirwany N.A., Zou M.H. (2014). AMPK: A cellular metabolic and redox sensor. A minireview. Front. Biosci..

[53] Yan H., Zhou H.F., Hu Y., Pham C.T. (2015). Suppression of experimental arthritis through AMP-activated protein kinase activation and autophagy modulation. J. Rheum. Dis. Treat..

[54] Thornton C.C., Al-Rashed F., Calay D., Birdsey G.M., Bauer A., Mylroie H., Morley B.J., Randi A.M., Haskard D.O., Boyle J.J. (2016). Methotrexate-mediated activation of an AMPK-CREB-dependent pathway: A novel mechanism for vascular protection in chronic systemic inflammation. Ann. Rheum. Dis..

[55] Wen Z., Jin K., Shen Y., Yang Z., Li Y., Wu B., Tian L., Shoor S., Roche N.E., Goronzy J.J. (2019). N-myristoyltransferase deficiency impairs activation of kinase AMPK and promotes synovial tissue inflammation. Nat. Immunol..

[56] Son H.J., Lee J., Lee S.Y., Kim E.K., Park M.J., Kim K.W., Park S.H., Cho M.L. (2014). Metformin attenuates experimental autoimmune arthritis through reciprocal regulation of Th17/Treg balance and osteoclastogenesis. Mediat. Inflamm..

[57] Pollizzi K.N., Powell J.D. (2015). Regulation of T cells by mTOR: The known knowns and the known unknowns. Trends Immunol..

[58] Pucino V., Lucherini O.M., Perna F., Obici L., Merlini G., Cattalini M., La Torre F., Maggio M.C., Lepore M.T., Magnotti F. (2016). Differential impact of high and low penetrance TNFRSF1A gene mutations on conventional and regulatory CD4+ T cell functions in TNFR1-associated periodic syndrome. J. Leukoc. Biol..

[59] Perl A. (2016). Activation of mTOR (mechanistic target of rapamycin) in rheumatic diseases. Nat. Rev. Rheumatol..

[60] Kuhnke A., Burmester G.R., Krauss S., Buttgereit F. (2003). Bioenergetics of immune cells to assess rheumatic disease activity and efficacy of glucocorticoid treatment. Ann. Rheum. Dis..

[61] Cronstein B.N., Aune T.M. (2020). Methotrexate and its mechanisms of action in inflammatory arthritis. Nat. Rev. Rheumatol..

[62] McGarry T., Orr C., Wade S., Biniecka M., Gallagher L., Low C., Veale D.J., Fearon U. (2018). JAK/STAT blockade alters synovial bioenergetics, mitochondrial function, and proinflammatory mediators in rheumatoid arthritis. Arthritis Rheumatol..

[63] Ruiz-Limón P., Ortega R., de la Rosa I.A., Abalos-Aguilera M.D.C., Perez-Sanchez C., Jimenez-Gomez Y., Peralbo-Santaella E., Font P., Ruiz-Vilches D., Ferrin G. (2017). Tocilizumab improves the proatherothrombotic profile of rheumatoid arthritis patients modulating endothelial dysfunction, NETosis, and inflammation. Transl. Res..

[64] Shen Y., Wen Z., Li Y., Matteson E.L., Hong J., Goronzy J.J., Weyand C.M. (2017). Metabolic control of the scaffold protein TKS5 in tissue-invasive, proinflammatory T cells. Nat. Immunol..

[65] Bustamante M.F., Oliveira P.G., Garcia-Carbonell R., Croft A.P., Smith J.M., Serrano R.L., Sanchez-Lopez E., Liu X., Kisseleva T., Hay N. (2018). Hexokinase 2 as a novel selective metabolic target for rheumatoid arthritis. Ann. Rheum. Dis..

[66] Choy E., Sattar N. (2009). Interpreting lipid levels in the context of high-grade inflammatory states with a focus on rheumatoid arthritis: A challenge to conventional cardiovascular risk actions. Ann. Rheum. Dis..

[67] Van Halm V.P., Nurmohamed M.T., Twisk J.W., Dijkmans B.A., Voskuyl A.E. (2006). Disease-modifying antirheumatic drugs are associated with a reduced risk for cardiovascular disease in patients with rheumatoid arthritis: A case control study. Arthritis Res. Ther..

[68] Dixon W.G., Watson K.D., Lunt M., Hyrich K.L., Silman A.J., Symmons D.P. (2007). Reduction in the incidence of myocardial infarction in patients with rheumatoid arthritis who respond to anti-tumor necrosis factor alpha therapy: Results from the British Society for Rheumatology Biologics Register. Arthritis Rheum..

[69] Greenberg J.D., Kremer J.M., Curtis J.R., Hochberg M.C., Reed G., Tsao P., Farkouh M.E., Nasir A., Setoguchi S., Solomon D.H. (2011). Tumour necrosis factor antagonist use and associated risk reduction of cardiovascular events among patients with rheumatoid arthritis. Ann. Rheum. Dis..

[70] Popa C., Netea M.G., Radstake T., Van der Meer J.W., Stalenhoef A.F., van Riel P.L., Barerra P. (2005). Influence of anti-tumour necrosis factor therapy on cardiovascular risk factors in patients with active rheumatoid arthritis. Ann. Rheum. Dis..

[71] Gossec L., Salejan F., Nataf H., Nguyen M., Gaud-Listrat V., Hudry C., Breuillard P., Dernis E., Boumier P., Durandin-Truffinet M. (2013). Challenges of cardiovascular risk assessment in the routine rheumatology outpatient setting: An observational study of 110 rheumatoid arthritis patients. Arthritis Care Res..

[72] Crowson C.S., Gabriel S.E. (2011). Towards improving cardiovascular risk management in patients with rheumatoid arthritis: The need for accurate risk assessment. Ann. Rheum. Dis..

[73] Arts E.E., Popa C., Den Broeder A.A., Semb A.G., Toms T., Kitas G.D., van Riel P.L., Fransen J. (2015). Performance of four current risk algorithms in predicting cardiovascular events in patients with early rheumatoid arthritis. Ann. Rheum. Dis..

[74] Jellinger P.S., Smith D.A., Mehta A.E., Ganda O., Handelsman Y., Rodbard H.W., Shepherd M.D., Seibel J.A. (2012). American Association of Clinical Endocrinologists’ Guidelines for Management of Dyslipidemia and Prevention of Atherosclerosis. Endocr. Pract..

[75] Libby P., Ridker P.M., Hansson G.K. (2011). Progress and challenges in translating the biology of atherosclerosis. Nature.

[76] Hansson G.K., Hermansson A. (2011). The immune system in atherosclerosis. Nat. Immunol..

[77] Danesh J., Kaptoge S., Mann A.G., Sarwar N., Wood A., Angleman S.B., Wensley F., Higgins J.P., Lennon L., Eiriksdottir G. (2008). Long-term interleukin-6 levels and subsequent risk of coronary heart disease: Two new prospective studies and a systematic review. PLoS Med..

[78] Kaptoge S., Di Angelantonio E., Lowe G., Pepys M.B., Thompson S.G., Collins R., Danesh J., Emerging Risk Factors Collaboration (2010). C-reactive protein concentration and risk of coronary heart disease, stroke, and mortality: An individual participant meta-analysis. Lancet.

[79] Danesh J., Lewington S., Thompson S.G., Lowe G.D., Collins R., Kostis J.B., Wilson A.C., Folsom A.R., Wu K., Fibrinogen Studies Collaboration (2005). Plasma fibrinogen level and the risk of major cardiovascular diseases and nonvascular mortality: An individual participant meta-analysis. JAMA.

[80] Schultz O., Oberhauser F., Saech J., Rubbert-Roth A., Hahn M., Krone W., Laudes M. (2010). Effects of inhibition of interleukin-6 signalling on insulin sensitivity and lipoprotein (a) levels in human subjects with rheumatoid diseases. PLoS ONE.

[81] Chung C.P., Oeser A., Solus J.F., Gebretsadik T., Shintani A., Avalos I., Sokka T., Raggi P., Pincus T., Stein C.M. (2008). Inflammation-associated insulin resistance: Differential effects in rheumatoid arthritis and systemic lupus erythematosus define potential mechanisms. Arthritis Rheum..

[82] Libby P. (2008). Role of inflammation in atherosclerosis associated with rheumatoid arthritis. Am. J. Med..

[83] Innala L., Möller B., Ljung L., Magnusson S., Smedby T., Södergren A., Öhman M.L., Rantapää-Dahlqvist S., Wållberg-Jonsson S. (2011). Cardiovascular events in early RA are a result of inflammatory burden and traditional risk factors: A five year prospective study. Arthritis Res. Ther..

[84] Book C., Saxne T., Jacobsson L.T. (2005). Prediction of mortality in rheumatoid arthritis based on disease activity markers. J. Rheumatol..

[85] Crilly M.A., Kumar V., Clark H.J., Scott N.W., Macdonald A.G., Williams D.J. (2009). Arterial stiffness and cumulative inflammatory burden in rheumatoid arthritis: A dose-response relationship independent of established cardiovascular risk factors. Rheumatology.

[86] Graf J., Scherzer R., Grunfeld C., Imboden J. (2009). Levels of C-reactive protein associated with high and very high cardiovascular risk are prevalent in patients with rheumatoid arthritis. PLoS ONE.

[87] Del Rincón I., Freeman G.L., Haas R.W., O’Leary D.H., Escalante A. (2005). Relative contribution of cardiovascular risk factors and rheumatoid arthritis clinical manifestations to atherosclerosis. Arthritis Rheum..

[88] Gonzalez-Gay M.A., Gonzalez-Juanatey C., Piñeiro A., Garcia-Porrua C., Testa A., Llorca J. (2005). High-grade C-reactive protein elevation correlates with accelerated atherogenesis in patients with rheumatoid arthritis. J. Rheumatol..

[89] Maradit-Kremers H., Nicola P.J., Crowson C.S., Ballman K.V., Gabriel S.E. (2005). Cardiovascular death in rheumatoid arthritis: A population-based study. Arthritis Rheum..

[90] Wållberg-Jonsson S., Johansson H., Ohman M.L., Rantapää-Dahlqvist S. (1999). Extent of inflammation predicts cardiovascular disease and overall mortality in seropositive rheumatoid arthritis. A retrospective cohort study from disease onset. J. Rheumatol..

[91] Miller A.M., McInnes I.B. (2011). Cytokines as therapeutic targets to reduce cardiovascular risk in chronic inflammation. Curr. Pharm. Des..

[92] Robertson J., Peters M.J., McInnes I.B., Sattar N. (2013). Changes in lipid levels with inflammation and therapy in RA: A maturing paradigm. Nat. Rev. Rheumatol..

[93] Akgün S., Ertel N.H., Mosenthal A., Oser W. (1998). Postsurgical reduction of serum lipoproteins: Interleukin-6 and the acute-phase response. J. Lab. Clin. Med..

[94] Daïen C.I., Duny Y., Barnetche T., Daurès J.P., Combe B., Morel J. (2012). Effect of TNF inhibitors on lipid profile in rheumatoid arthritis: A systematic review with meta-analysis. Ann. Rheum. Dis..

[95] Singh U., Dasu M.R., Yancey P.G., Afify A., Devaraj S., Jialal I. (2008). Human C-reactive protein promotes oxidized low density lipoprotein uptake and matrix metalloproteinase-9 release in Wistar rats. J. Lipid. Res..

[96] Wang X., Liao D., Bharadwaj U., Li M., Yao Q., Chen C. (2008). C-reactive protein inhibits cholesterol efflux from human macrophage-derived foam cells. Arter. Thromb. Vasc. Biol..

[97] Watanabe J., Charles-Schoeman C., Miao Y., Elashoff D., Lee Y.Y., Katselis G., Lee T.D., Reddy S.T. (2012). Proteomic profiling following immunoaffinity capture of high-density lipoprotein: Association of acute-phase proteins and complement factors with proinflammatory high-density lipoprotein in rheumatoid arthritis. Arthritis Rheum..

[98] Berrougui H., Momo C.N., Khalil A. (2012). Health benefits of high-density lipoproteins in preventing cardiovascular diseases. J. Clin. Lipidol..

[99] Charles-Schoeman C., Lee Y.Y., Grijalva V., Amjadi S., FitzGerald J., Ranganath V.K., Taylor M., McMahon M., Paulus H.E., Reddy S.T. (2012). Cholesterol efflux by high density lipoproteins is impaired in patients with active rheumatoid arthritis. Ann. Rheum. Dis..

[100] Watanabe J., Chou K.J., Liao J.C., Miao Y., Meng H.H., Ge H., Grijalva V., Hama S., Kozak K., Buga G. (2007). Differential association of hemoglobin with proinflammatory high density lipoproteins in atherogenic/hyperlipidemic mice. A novel biomarker of atherosclerosis. J. Biol. Chem..

[101] Mackness M.I., Durrington P.N., Mackness B. (2004). The role of paraoxonase 1 activity in cardiovascular disease: Potential for therapeutic intervention. Am. J. Cardiovasc. Drugs.

[102] Navab M., Berliner J.A., Subbanagounder G., Hama S., Lusis A.J., Castellani L.W., Reddy S., Shih D., Shi W., Watson A.D. (2001). HDL and the inflammatory response induced by LDL-derived oxidized phospholipids. Arter. Thromb. Vasc. Biol..

[103] Van Lenten B.J., Wagner A.C., Nayak D.P., Hama S., Navab M., Fogelman A.M. (2001). High-density lipoprotein loses its anti-inflammatory properties during acute influenza a infection. Circulation.

[104] Van Lenten B.J., Hama S.Y., de Beer F.C., Stafforini D.M., McIntyre T.M., Prescott S.M., La Du B.N., Fogelman A.M., Navab M. (1995). Anti-inflammatory HDL becomes pro-inflammatory during the acute phase response. Loss of protective effect of HDL against LDL oxidation in aortic wall cell cocultures. J. Clin. Investig..

[105] Raterman H.G., Levels H., Voskuyl A.E., Lems W.F., Dijkmans B.A., Nurmohamed M.T. (2013). HDL protein composition alters from proatherogenic into less atherogenic and proinflammatory in rheumatoid arthritis patients responding to rituximab. Ann. Rheum. Dis..

[106] Jamnitski A., Levels J.H., van den Oever I.A., Nurmohamed M.T. (2013). High-density lipoprotein profiling changes in patients with rheumatoid arthritis treated with tumor necrosis factor inhibitors: A cohort study. J. Rheumatol..

[107] Schwartz G.G., Olsson A.G., Abt M., Ballantyne C.M., Barter P.J., Brumm J., Chaitman B.R., Holme I.M., Kallend D., Leiter L.A. (2012). Effects of dalcetrapib in patients with a recent acute coronary syndrome. N. Engl. J. Med..

[108] Zhao L., Jin W., Rader D., Packard C., Feuerstein G. (2009). A Translational Medicine perspective of the development of torcetrapib: Does the failure of torcetrapib development cast a shadow on future development of lipid modifying agents, HDL elevation strategies or CETP as a viable molecular target for atherosclerosis? A case study of the use of biomarkers and Translational Medicine in atherosclerosis drug discovery and development. Biochem. Pharmacol..

[109] Navab M., Hama S.Y., Cooke C.J., Anantharamaiah G.M., Chaddha M., Jin L., Subbanagounder G., Faull K.F., Reddy S.T., Miller N.E. (2000). Normal high density lipoprotein inhibits three steps in the formation of mildly oxidized low density lipoprotein: Step 1. J. Lipid. Res..

[110] Navab M., Hama S.Y., Anantharamaiah G.M., Hassan K., Hough G.P., Watson A.D., Reddy S.T., Sevanian A., Fonarow G.C., Fogelman A.M. (2000). Normal high density lipoprotein inhibits three steps in the formation of mildly oxidized low density lipoprotein: Steps 2 and 3. J. Lipid. Res..

[111] Ansell B.J., Navab M., Hama S., Kamranpour N., Fonarow G., Hough G., Rahmani S., Mottahedeh R., Dave R., Reddy S.T. (2003). Inflammatory/antiinflammatory properties of high-density lipoprotein distinguish patients from control subjects better than high-density lipoprotein cholesterol levels and are favorably affected by simvastatin treatment. Circulation.

[112] Venetsanopoulou A.I., Pelechas E., Voulgari P.V., Drosos A.A. (2020). The lipid paradox in rheumatoid arthritis: The dark horse of the augmented cardiovascular risk. Rheumatol. Int..

[113] Makkar R., Behl T., Kumar A., Uddin M.S., Bungau S. (2020). Untying the correlation between apolipoproteins and rheumatoid arthritis. Inflamm. Res..

[114] Keller R.K., Enna S.J., Bylund D.B. (2007). Lipids. xPharm: The Comprehensive Pharmacology Reference.

[115] Feingold K.R., Grunfeld C., Feingold K.R., Anawalt B., Boyce A., Chrousos G., de Herder W.W., Dungan K., Grossman A., Hershman J.M., Hofland H.J., Kaltsas G. (2018). Introduction to Lipids and Lipoproteins. Endotext.

[116] Kaur I., Behl T., Bungau S., Zengin G., Kumar A., El-Esawi M.A., Khullar G., Venkatachalam T., Arora S. (2020). The endocannabinoid signaling pathway as an emerging target in pharmacotherapy, earmarking mitigation of destructive events in rheumatoid arthritis. Life Sci..

[117] Kaur I., Behl T., Bungau S., Kumar A., Mehta V., Setia D., Uddin S., Zengin G., Aleya L., Arora S. (2020). Exploring the therapeutic promise of targeting HMGB1 in rheumatoid arthritis. Life Sci..

[118] Mahley R.W., Innerarity T.L., Rall S.C., Weisgraber K.H. (1984). Plasma lipoproteins: Apolipoprotein structure and function. J. Lipid. Res..

[119] Canbay A., Bechmann L., Gerken G. (2007). Lipid metabolism in the liver. Z. Gastroenterol..

[120] Nguyen P., Leray V., Diez M., Serisier S., Le Bloc’h J., Siliart B., Dumon H. (2008). Liver lipid metabolism. J. Anim. Physiol. Anim. Nutr..

[121] Brouwers H., von Hegedus J., Toes R., Kloppenburg M., Ioan-Facsinay A. (2015). Lipid mediators of inflammation in rheumatoid arthritis and osteoarthritis. Best Pract. Res. Clin. Rheumatol..

[122] Rodríguez-Carrio J., Coras R., Alperi-López M., López P., Ulloa C., Ballina-García F.J., Armando A.M., Quehenberger O., Guma M., Suárez A. (2020). Serum oxylipins profiling during the earliest stages of rheumatoid arthritis. Arthritis Rheumatol..

[123] Quehenberger O., Armando A.M., Brown A.H., Milne S.B., Myers D.S., Merrill A.H., Bandyopadhyay S., Jones K.N., Kelly S., Shaner R.L. (2010). Lipidomics reveals a remarkable diversity of lipids in human plasma. J. Lipid. Res..

[124] Miles E.A., Calder P.C. (2012). Influence of marine n-3 polyunsaturated fatty acids on immune function and a systematic review of their effects on clinical outcomes in rheumatoid arthritis. Br. J. Nutr..

[125] Ormseth M.J., Swift L.L., Fazio S., Linton M.F., Chung C.P., Raggi P., Rho Y.H., Solus J., Oeser A., Bian A. (2011). Free fatty acids are associated with insulin resistance but not coronary artery atherosclerosis in rheumatoid arthritis. Atherosclerosis.

[126] Fuchs B., Schiller J., Wagner U., Häntzschel H., Arnold K. (2005). The phosphatidylcholine/lysophosphatidylcholine ratio in human plasma is an indicator of the severity of rheumatoid arthritis: Investigations by 31P NMR and MALDI-TOF MS. Clin. Biochem..

[127] Moghaddami M., Ranieri E., James M., Fletcher J., Cleland L.G. (2013). Prostaglandin D(2) in inflammatory arthritis and its relation with synovial fluid dendritic cells. Mediat. Inflamm..

[128] Kosinska M.K., Liebisch G., Lochnit G., Wilhelm J., Klein H., Kaesser U., Lasczkowski G., Rickert M., Schmitz G., Steinmeyer J. (2013). A lipidomic study of phospholipid classes and species in human synovial fluid. Arthritis Rheum..

[129] Korotkova M., Jakobsson P.J. (2014). Persisting eicosanoid pathways in rheumatic diseases. Nat. Rev. Rheumatol..

[130] Pruzanski W., Vadas P., Kim J., Jacobs H., Stefanski E. (1988). Phospholipase A2 activity associated with synovial fluid cells. J. Rheumatol..

[131] Giera M., Ioan-Facsinay A., Toes R., Gao F., Dalli J., Deelder A.M., Serhan C.N., Mayboroda O.A. (2012). Lipid and lipid mediator profiling of human synovial fluid in rheumatoid arthritis patients by means of LC-MS/MS. Biochim. Biophys. Acta.

[132] Serhan C.N., Chiang N., Dalli J. (2015). The resolution code of acute inflammation: Novel pro-resolving lipid mediators in resolution. Semin. Immunol..

[133] Georgiadis A.N., Papavasiliou E.C., Lourida E.S., Alamanos Y., Kostara C., Tselepis A.D., Drosos A.A. (2006). Atherogenic lipid profile is a feature characteristic of patients with early rheumatoid arthritis: Effect of early treatment--a prospective, controlled study. Arthritis Res. Ther..

[134] Georgiadis A.N., Voulgari P.V., Argyropoulou M.I., Alamanos Y., Elisaf M., Tselepis A.D., Drosos A.A. (2008). Early treatment reduces the cardiovascular risk factors in newly diagnosed rheumatoid arthritis patients. Semin. Arthritis Rheum..

[135] Ridker P.M., Danielson E., Fonseca F.A., Genest J., Gotto A.M., Kastelein J.J., Koenig W., Libby P., Lorenzatti A.J., Macfadyen J.G. (2009). Reduction in C-reactive protein and LDL cholesterol and cardiovascular event rates after initiation of rosuvastatin: A prospective study of the JUPITER trial. Lancet.

[136] Van Lenten B.J., Reddy S.T., Navab M., Fogelman A.M. (2006). Understanding changes in high density lipoproteins during the acute phase response. Arter. Thromb. Vasc. Biol..

[137] Navarro-Millán I., Charles-Schoeman C., Yang S., Bathon J.M., Bridges S.L., Chen L., Cofield S.S., Dell’Italia L.J., Moreland L.W., O’Dell J.R. (2013). Changes in lipoproteins associated with methotrexate or combination therapy in early rheumatoid arthritis: Results from the treatment of early rheumatoid arthritis trial. Arthritis Rheum..

[138] Liao K.P., Playford M.P., Frits M., Coblyn J.S., Iannaccone C., Weinblatt M.E., Shadick N.S., Mehta N.N. (2015). The association between reduction in inflammation and changes in lipoprotein levels and HDL cholesterol efflux capacity in rheumatoid arthritis. J. Am. Heart Assoc..

[139] Hashizume M., Mihara M. (2012). Atherogenic effects of TNF-α and IL-6 via up-regulation of scavenger receptors. Cytokine.

[140] Lubrano V., Gabriele M., Puntoni M.R., Longo V., Pucci L. (2015). Relationship among IL-6, LDL cholesterol and lipid peroxidation. Cell. Mol. Biol. Lett..

[141] Umpleby A.M. (2015). Hormone measurement guidelines: Tracing lipid metabolism: The value of stable isotopes. J. Endocrinol..

[142] Charles-Schoeman C., Fleischmann R., Davignon J., Schwartz H., Turner S.M., Beysen C., Milad M., Hellerstein M.K., Luo Z., Kaplan I.V. (2015). Potential mechanisms leading to the abnormal lipid profile in patients with rheumatoid arthritis versus healthy volunteers and reversal by tofacitinib. Arthritis Rheumatol..

[143] Robertson J., Porter D., Sattar N., Packard C.J., Caslake M., McInnes I., McCarey D. (2017). Interleukin-6 blockade raises LDL via reduced catabolism rather than via increased synthesis: A cytokine-specific mechanism for cholesterol changes in rheumatoid arthritis. Ann. Rheum. Dis..

[144] Chistiakov D.A., Bobryshev Y.V., Orekhov A.N. (2016). Macrophage-mediated cholesterol handling in atherosclerosis. J. Cell. Mol. Med..

[145] Lourida E.S., Georgiadis A.N., Papavasiliou E.C., Papathanasiou A.I., Drosos A.A., Tselepis A.D. (2007). Patients with early rheumatoid arthritis exhibit elevated autoantibody titers against mildly oxidized low-density lipoprotein and exhibit decreased activity of the lipoprotein-associated phospholipase A2. Arthritis Res. Ther..

[146] Toms T.E., Panoulas V.F., Douglas K.M., Nightingale P., Smith J.P., Griffiths H., Sattar N., Symmons D.P., Kitas G.D. (2011). Are lipid ratios less susceptible to change with systemic inflammation than individual lipid components in patients with rheumatoid arthritis?. Angiology.

[147] Del Rincón I., Polak J.F., O’Leary D.H., Battafarano D.F., Erikson J.M., Restrepo J.F., Molina E., Escalante A. (2015). Systemic inflammation and cardiovascular risk factors predict rapid progression of atherosclerosis in rheumatoid arthritis. Ann. Rheum. Dis..

[148] Ambrosino P., Lupoli R., Di Minno A., Tasso M., Peluso R., Di Minno M.N. (2015). Subclinical atherosclerosis in patients with rheumatoid arthritis. A meta-analysis of literature studies. Thromb. Haemost..

[149] Ridker P.M., Everett B.M., Thuren T., MacFadyen J.G., Chang W.H., Ballantyne C., Fonseca F., Nicolau J., Koenig W., Anker S.D. (2017). Antiinflammatory therapy with canakinumab for atherosclerotic disease. N. Engl. J. Med..

[150] Baker J.D. (2016). The purpose, process, and methods of writing a literature review. AORN J..

[151] Hayek T., Oiknine J., Brook J.G., Aviram M. (1994). Role of HDL apolipoprotein E in cellular cholesterol efflux: Studies in apo E knockout transgenic mice. Biochem. Biophys. Res. Commun..

[152] Navab M., Ananthramaiah G.M., Reddy S.T., Van Lenten B.J., Ansell B.J., Hama S., Hough G., Bachini E., Grijalva V.R., Wagner A.C. (2005). The double jeopardy of HDL. Ann. Med..

[153] Popa C., van Tits L.J., Barrera P., Lemmers H.L., van den Hoogen F.H., van Riel P.L., Radstake T.R., Netea M.G., Roest M., Stalenhoef A.F. (2009). Anti-inflammatory therapy with tumour necrosis factor alpha inhibitors improves high-density lipoprotein cholesterol antioxidative capacity in rheumatoid arthritis patients. Ann. Rheum. Dis..

[154] McInnes I.B., Thompson L., Giles J.T., Bathon J.M., Salmon J.E., Beaulieu A.D., Codding C.E., Carlson T.H., Delles C., Lee J.S. (2015). Effect of interleukin-6 receptor blockade on surrogates of vascular risk in rheumatoid arthritis: MEASURE, a randomised, placebo-controlled study. Ann. Rheum. Dis..

[155] Park J., Kim M., Kang S.G., Jannasch A.H., Cooper B., Patterson J., Kim C.H. (2015). Short-chain fatty acids induce both effector and regulatory T cells by suppression of histone deacetylases and regulation of the mTOR-S6K pathway. Mucosal. Immunol..

[156] Cipolletta D., Feuerer M., Li A., Kamei N., Lee J., Shoelson S.E., Benoist C., Mathis D. (2012). PPAR-γ is a major driver of the accumulation and phenotype of adipose tissue Treg cells. Nature.

[157] Kidani Y., Elsaesser H., Hock M.B., Vergnes L., Williams K.J., Argus J.P., Marbois B.N., Komisopoulou E., Wilson E.B., Osborne T.F. (2013). Sterol regulatory element-binding proteins are essential for the metabolic programming of effector T cells and adaptive immunity. Nat. Immunol..

[158] Yang Z., Fujii H., Mohan S.V., Goronzy J.J., Weyand C.M. (2013). Phosphofructokinase deficiency impairs ATP generation, autophagy, and redox balance in rheumatoid arthritis T cells. J. Exp. Med..

[159] Berod L., Friedrich C., Nandan A., Freitag J., Hagemann S., Harmrolfs K., Sandouk A., Hesse C., Castro C.N., Bähre H. (2014). De novo fatty acid synthesis controls the fate between regulatory T and T helper 17 cells. Nat. Med..

[160] Pucino V., Certo M., Bulusu V., Cucchi D., Goldmann K., Pontarini E., Haas R., Smith J., Headland S.E., Blighe K. (2019). Lactate buildup at the site of chronic inflammation promotes disease by inducing CD4(+) T cell metabolic rewiring. Cell Metab..

[161] Perucha E., Melchiotti R., Bibby J.A., Wu W., Frederiksen K.S., Roberts C.A., Hall Z., LeFriec G., Robertson K.A., Lavender P. (2019). The cholesterol biosynthesis pathway regulates IL-10 expression in human Th1 cells. Nat. Commun..

[162] Ahn J.K., Kim S., Hwang J., Kim J., Kim K.H., Cha H.S. (2016). GC/TOF-MS-based metabolomic profiling in cultured fibroblast-like synoviocytes from rheumatoid arthritis. Jt. Bone Spine.

[163] Volchenkov R., Cao M.D., Elgstøen K.B., Goll G.L., Eikvar K., Bjørneboe O., Bathen T.F., Holen H.L., Kvien T.K., Skålhegg B.S. (2017). Metabolic profiling of synovial tissue shows altered glucose and choline metabolism in rheumatoid arthritis samples. Scand. J. Rheumatol..

[164] Seki M., Kawai Y., Ishii C., Yamanaka T., Odawara M., Inazu M. (2017). Functional analysis of choline transporters in rheumatoid arthritis synovial fibroblasts. Mod. Rheumatol..

[165] Chen D.Y., Chen Y.M., Hsieh T.Y., Hsieh C.W., Lin C.C., Lan J.L. (2015). Significant effects of biologic therapy on lipid profiles and insulin resistance in patients with rheumatoid arthritis. Arthritis Res. Ther..

[166] McGrath C.M., Young S.P. (2015). Lipid and metabolic changes in rheumatoid arthritis. Curr. Rheumatol. Rep..

[167] Marks J.L., Edwards C.J. (2012). Protective effect of methotrexate in patients with rheumatoid arthritis and cardiovascular comorbidity. Ther. Adv. Musculoskelet. Dis..

[168] Morris S.J., Wasko M.C., Antohe J.L., Sartorius J.A., Kirchner H.L., Dancea S., Bili A. (2011). Hydroxychloroquine use associated with improvement in lipid profiles in rheumatoid arthritis patients. Arthritis Care Res..

[169] Toussirot É. (2015). Effects of TNFα inhibitors on adiposity and other cardiovascular risk factors: Implications for the cardiovascular prognosis in patients with rheumatoid arthritis. Expert Opin. Drug Saf..

[170] Chung C.P., Giles J.T., Petri M., Szklo M., Post W., Blumenthal R.S., Gelber A.C., Ouyang P., Jenny N.S., Bathon J.M. (2012). Prevalence of traditional modifiable cardiovascular risk factors in patients with rheumatoid arthritis: Comparison with control subjects from the multi-ethnic study of atherosclerosis. Semin. Arthritis Rheum..

[171] Vesa C.M., Popa L., Popa A.R., Rus M., Zaha A.A., Bungau S., Tit D.M., Aron R.A.C., Zaha D.C. (2020). Current Data Regarding the Relationship between Type 2 Diabetes Mellitus and Cardiovascular Risk Factors. Diagnostics.

[172] Davis J.M., Kremers H.M., Crowson C.S., Nicola P.J., Ballman K.V., Therneau T.M., Roger V.L., Gabriel S.E. (2007). Glucocorticoids and cardiovascular events in rheumatoid arthritis: A population-based cohort study. Arthritis Rheum..

[173] Zampeli E., Protogerou A., Stamatelopoulos K., Fragiadaki K., Katsiari C.G., Kyrkou K., Papamichael C.M., Mavrikakis M., Nightingale P., Kitas G.D. (2012). Predictors of new atherosclerotic carotid plaque development in patients with rheumatoid arthritis: A longitudinal study. Arthritis Res. Ther..

[174] Hafström I., Rohani M., Deneberg S., Wörnert M., Jogestrand T., Frostegård J. (2007). Effects of low-dose prednisolone on endothelial function, atherosclerosis, and traditional risk factors for atherosclerosis in patients with rheumatoid arthritis—A randomized study. J. Rheumatol..

[175] Micha R., Imamura F., von Ballmoos M.W., Solomon D.H., Hernán M.A., Ridker P.M., Mozaffarian D. (2011). Systematic review and meta-analysis of methotrexate use and risk of cardiovascular disease. Am. J. Cardiol..

[176] Reiss A.B., Carsons S.E., Anwar K., Rao S., Edelman S.D., Zhang H., Fernandez P., Cronstein B.N., Chan E.S. (2008). Atheroprotective effects of methotrexate on reverse cholesterol transport proteins and foam cell transformation in human THP-1 monocyte/macrophages. Arthritis Rheum..

[177] Rho Y.H., Oeser A., Chung C.P., Milne G.L., Stein C.M. (2009). Drugs used in the treatment of rheumatoid arthritis: Relationship between current use and cardiovascular risk factors. Arch. Drug Inf..

[178] Ormseth M.J., Yancey P.G., Solus J.F., Bridges S.L., Curtis J.R., Linton M.F., Fazio S., Davies S.S., Roberts L.J., Vickers K.C. (2016). Effect of drug therapy on net cholesterol efflux capacity of high-density lipoprotein-enriched serum in rheumatoid arthritis. Arthritis Rheumatol..

[179] O’Neill F., Charakida M., Topham E., McLoughlin E., Patel N., Sutill E., Kay C.W.M., D’Aiuto F., Landmesser U., Taylor P.C. (2017). Anti-inflammatory treatment improves high-density lipoprotein function in rheumatoid arthritis. Heart.

[180] Park Y.B., Choi H.K., Kim M.Y., Lee W.K., Song J., Kim D.K., Lee S.K. (2002). Effects of antirheumatic therapy on serum lipid levels in patients with rheumatoid arthritis: A prospective study. Am. J. Med..

[181] Ronda N., Greco D., Adorni M.P., Zimetti F., Favari E., Hjeltnes G., Mikkelsen K., Borghi M.O., Favalli E.G., Gatti R. (2015). Newly identified antiatherosclerotic activity of methotrexate and adalimumab: Complementary effects on lipoprotein function and macrophage cholesterol metabolism. Arthritis Rheumatol..

[182] Rodriguez-Jimenez N.A., Garcia-Gonzalez C.E., Ayala-Lopez K.P., Trujillo-Hernandez B., Aguilar-Chavez E.A., Rocha-Muñoz A.D., Vasquez-Jimenez J.C., Olivas-Flores E., Salazar-Paramo M., Corona-Sanchez E.G. (2014). Modifications in lipid levels are independent of serum TNF-α in rheumatoid arthritis: Results of an observational 24-week cohort study comparing patients receiving etanercept plus methotrexate or methotrexate as monotherapy. Biomed. Res. Int..

[183] Tracey D., Klareskog L., Sasso E.H., Salfeld J.G., Tak P.P. (2008). Tumor necrosis factor antagonist mechanisms of action: A comprehensive review. Pharmacol. Ther..

[184] Stagakis I., Bertsias G., Karvounaris S., Kavousanaki M., Virla D., Raptopoulou A., Kardassis D., Boumpas D.T., Sidiropoulos P.I. (2012). Anti-tumor necrosis factor therapy improves insulin resistance, beta cell function and insulin signaling in active rheumatoid arthritis patients with high insulin resistance. Arthritis Res. Ther..

[185] Wijbrandts C.A., van Leuven S.I., Boom H.D., Gerlag D.M., Stroes E.G., Kastelein J.J., Tak P.P. (2009). Sustained changes in lipid profile and macrophage migration inhibitory factor levels after anti-tumour necrosis factor therapy in rheumatoid arthritis. Ann. Rheum. Dis..

[186] Sattar N., Crompton P., Cherry L., Kane D., Lowe G., McInnes I.B. (2007). Effects of tumor necrosis factor blockade on cardiovascular risk factors in psoriatic arthritis: A double-blind, placebo-controlled study. Arthritis Rheum..

[187] Barnabe C., Martin B.J., Ghali W.A. (2011). Systematic review and meta-analysis: Anti-tumor necrosis factor α therapy and cardiovascular events in rheumatoid arthritis. Arthritis Care Res..

[188] Wong M., Oakley S.P., Young L., Jiang B.Y., Wierzbicki A., Panayi G., Chowienczyk P., Kirkham B. (2009). Infliximab improves vascular stiffness in patients with rheumatoid arthritis. Ann. Rheum. Dis..

[189] Hürlimann D., Forster A., Noll G., Enseleit F., Chenevard R., Distler O., Béchir M., Spieker L.E., Neidhart M., Michel B.A. (2002). Anti-tumor necrosis factor-alpha treatment improves endothelial function in patients with rheumatoid arthritis. Circulation.

[190] McKellar G.E., McCarey D.W., Sattar N., McInnes I.B. (2009). Role for TNF in atherosclerosis? Lessons from autoimmune disease. Nat. Rev. Cardiol..

[191] Del Porto F., Laganà B., Lai S., Nofroni I., Tinti F., Vitale M., Podestà E., Mitterhofer A.P., D’Amelio R. (2007). Response to anti-tumour necrosis factor alpha blockade is associated with reduction of carotid intima-media thickness in patients with active rheumatoid arthritis. Rheumatology.

[192] Popa C., van den Hoogen F.H., Radstake T.R., Netea M.G., Eijsbouts A.E., den Heijer M., van der Meer J.W., van Riel P.L., Stalenhoef A.F., Barrera P. (2007). Modulation of lipoprotein plasma concentrations during long-term anti-TNF therapy in patients with active rheumatoid arthritis. Ann. Rheum. Dis..

[193] Kiortsis D.N., Mavridis A.K., Filippatos T.D., Vasakos S., Nikas S.N., Drosos A.A. (2006). Effects of infliximab treatment on lipoprotein profile in patients with rheumatoid arthritis and ankylosing spondylitis. J. Rheumatol..

[194] Bergström U., Jovinge S., Persson J., Jacobsson L.T.H., Turesson C. (2018). Effects of treatment with adalimumab on blood lipid levels and atherosclerosis in patients with rheumatoid arthritis. Curr. Ther. Res. Clin. Exp..

[195] Seriolo B., Paolino S., Sulli A., Fasciolo D., Cutolo M. (2006). Effects of anti-TNF-alpha treatment on lipid profile in patients with active rheumatoid arthritis. Ann. N. Y. Acad. Sci..

[196] Van Sijl A.M., Peters M.J., Knol D.L., de Vet R.H., Sattar N., Dijkmans B.A., Smulders Y.M., Nurmohamed M.T. (2011). The effect of TNF-alpha blocking therapy on lipid levels in rheumatoid arthritis: A meta-analysis. Semin. Arthritis Rheum..

[197] Di Minno M.N., Ambrosino P., Peluso R., Di Minno A., Lupoli R., Dentali F., CaRRDs Study Group (2014). Lipid profile changes in patients with rheumatic diseases receiving a treatment with TNF-α blockers: A meta-analysis of prospective studies. Ann. Med..

[198] Cacciapaglia F., Anelli M.G., Rinaldi A., Serafino L., Covelli M., Scioscia C., Iannone F., Lapadula G. (2014). Lipid profile of rheumatoid arthritis patients treated with anti-tumor necrosis factor-alpha drugs changes according to disease activity and predicts clinical response. Drug Dev. Res..

[199] Végh E., Kerekes G., Pusztai A., Hamar A., Szamosi S., Váncsa A., Bodoki L., Pogácsás L., Balázs F., Hodosi K. (2020). Effects of 1-year anti-TNF-α therapy on vascular function in rheumatoid arthritis and ankylosing spondylitis. Rheumatol. Int..

[200] Kiortsis D.N., Mavridis A.K., Vasakos S., Nikas S.N., Drosos A.A. (2005). Effects of infliximab treatment on insulin resistance in patients with rheumatoid arthritis and ankylosing spondylitis. Ann. Rheum. Dis..

[201] Ljung L., Askling J., Rantapää-Dahlqvist S., Jacobsson L. (2014). The risk of acute coronary syndrome in rheumatoid arthritis in relation to tumour necrosis factor inhibitors and the risk in the general population: A national cohort study. Arthritis Res. Ther..

[202] De Sanctis S., Marcovecchio M.L., Gaspari S., Del Torto M., Mohn A., Chiarelli F., Breda L. (2013). Etanercept improves lipid profile and oxidative stress measures in patients with juvenile idiopathic arthritis. J. Rheumatol..

[203] Hashizume M., Yoshida H., Koike N., Suzuki M., Mihara M. (2010). Overproduced interleukin 6 decreases blood lipid levels via upregulation of very-low-density lipoprotein receptor. Ann. Rheum. Dis..

[204] Cacciapaglia F., Anelli M.G., Rinaldi A., Fornaro M., Lopalco G., Scioscia C., Lapadula G., Iannone F. (2018). Lipids and atherogenic indices fluctuation in rheumatoid arthritis patients on long-term tocilizumab treatment. Mediat. Inflamm..

[205] Kawashiri S.Y., Kawakami A., Yamasaki S., Imazato T., Iwamoto N., Fujikawa K., Aramaki T., Tamai M., Nakamura H., Ida H. (2011). Effects of the anti-interleukin-6 receptor antibody, tocilizumab, on serum lipid levels in patients with rheumatoid arthritis. Rheumatol. Int..

[206] Gabay C., Emery P., van Vollenhoven R., Dikranian A., Alten R., Pavelka K., Klearman M., Musselman D., Agarwal S., Green J. (2013). Tocilizumab monotherapy versus adalimumab monotherapy for treatment of rheumatoid arthritis (ADACTA): A randomised, double-blind, controlled phase 4 trial. Lancet.

[207] Gruzdeva O., Uchasova E., Dyleva Y., Belik E., Shurygina E., Barbarash O. (2013). Insulin resistance and inflammation markers in myocardial infarction. J. Inflamm. Res..

[208] Mirjafari H., Wang J., Klearman M., Harari O., Bruce I. (2014). FRI0132 Insulin resistance is improved by tocilizumab therapy in rheumatoid arthritis: Results from the toward study. Ann. Rheum. Dis..

[209] Schiff M.H., Kremer J.M., Jahreis A., Vernon E., Isaacs J.D., van Vollenhoven R.F. (2011). Integrated safety in tocilizumab clinical trials. Arthritis Res. Ther..

[210] Haraoui B., Sebba A., Rubbert-Roth A., Scali J., Alten R., Kremer J., Pitts L., Vernon E., van Vollenhoven R., Genovese M. (2014). Long-term safety of tocilizumab in patients with rheumatoid arthritis following a mean treatment duration of 3.9 years. Ann. Rheum. Dis..

[211] Fleischmann R., Kremer J., Cush J., Schulze-Koops H., Connell C.A., Bradley J.D., Gruben D., Wallenstein G.V., Zwillich S.H., Kanik K.S. (2012). Placebo-controlled trial of tofacitinib monotherapy in rheumatoid arthritis. N. Engl. J. Med..

[212] Charles-Schoeman C., Gonzalez-Gay M.A., Kaplan I., Boy M., Geier J., Luo Z., Zuckerman A., Riese R. (2016). Effects of tofacitinib and other DMARDs on lipid profiles in rheumatoid arthritis: Implications for the rheumatologist. Semin. Arthritis Rheum..

[213] Van Vollenhoven R.F., Fleischmann R., Cohen S., Lee E.B., García Meijide J.A., Wagner S., Forejtova S., Zwillich S.H., Gruben D., Koncz T. (2012). Tofacitinib or adalimumab versus placebo in rheumatoid arthritis. N. Engl. J. Med..

[214] McInnes I.B., Kim H.Y., Lee S.H., Mandel D., Song Y.W., Connell C.A., Luo Z., Brosnan M.J., Zuckerman A., Zwillich S.H. (2014). Open-label tofacitinib and double-blind atorvastatin in rheumatoid arthritis patients: A randomised study. Ann. Rheum. Dis..

[215] Novikova D.S., Popkova T.V., Lukina G.V., Luchikhina E.L., Karateev D.E., Volkov A.V., Novikov A.A., Aleksandrova E.N., Nasonov E.L. (2016). The effects of rituximab on lipids, arterial stiffness and carotid intima-media thickness in rheumatoid arthritis. J. Korean Med. Sci..

[216] Kerekes G., Soltész P., Dér H., Veres K., Szabó Z., Végvári A., Szegedi G., Shoenfeld Y., Szekanecz Z. (2009). Effects of rituximab treatment on endothelial dysfunction, carotid atherosclerosis, and lipid profile in rheumatoid arthritis. Clin. Rheumatol..

[217] Mathieu S., Pereira B., Dubost J.J., Lusson J.R., Soubrier M. (2012). No significant change in arterial stiffness in RA after 6 months and 1 year of rituximab treatment. Rheumatology.

[218] Ridker P.M., Cook N.R. (2013). Statins: New American guidelines for prevention of cardiovascular disease. Lancet.

[219] Kitas G.D., Nightingale P., Armitage J., Sattar N., Belch J.J.F., Symmons D.P.M., TRACE RA Consortium (2019). A multicenter, randomized, placebo-controlled trial of atorvastatin for the primary prevention of cardiovascular events in patients with rheumatoid arthritis. Arthritis Rheumatol..

[220] An J., Alemao E., Reynolds K., Kawabata H., Solomon D.H., Liao K.P., Niu F., Cheetham T.C. (2016). Cardiovascular outcomes associated with lowering low-density lipoprotein cholesterol in rheumatoid arthritis and matched nonrheumatoid arthritis. J. Rheumatol..

[221] Jafri K., Taylor L., Nezamzadeh M., Baker J.F., Mehta N.N., Bartels C., Williams C.T., Ogdie A. (2015). Management of hyperlipidemia among patients with rheumatoid arthritis in the primary care setting. BMC Musculoskelet. Disord..

